# AUXIN RESPONSE FACTOR 18–HISTONE DEACETYLASE 6 module regulates floral organ identity in rose (*Rosa hybrida*)

**DOI:** 10.1093/plphys/kiab130

**Published:** 2021-03-17

**Authors:** Jiwei Chen, Yang Li, Yonghong Li, Yuqi Li, Yi Wang, Chuyan Jiang, Patrick Choisy, Tao Xu, Youming Cai, Dong Pei, Cai-Zhong Jiang, Su-Sheng Gan, Junping Gao, Nan Ma

**Affiliations:** 1 State Key Laboratory of Agrobiotechnology, Beijing Key Laboratory of Development and Quality Control of Ornamental Crops, Department of Ornamental Horticulture, College of Horticulture, China Agricultural University, Beijing 100193, China; 2 School of Applied Chemistry and Biotechnology, Shenzhen Polytechnic, Shenzhen, Guangdong 518055, China; 3 LVMH Recherche, F-45800 St Jean de Braye, France; 4 Shanghai Academy of Agricultural Sciences, Shanghai, 201403, China; 5 State Key Laboratory of Tree Genetics and Breeding, Research Institute of Forestry, Chinese Academy of Forestry, Beijing 100091, China; 6 Crop Pathology and Genetic Research Unit, US Department of Agriculture, Agricultural Research Service, University of California, Davis, California, USA; 7 Department of Plant Sciences, University of California, Davis, California, USA; 8 Plant Biology Section, School of Integrative Plant Science, College of Agriculture and Life Sciences, Cornell University, Ithaca, New York, USA

## Abstract

The phytohormone auxin plays a pivotal role in floral meristem initiation and gynoecium development, but whether and how auxin controls floral organ identity remain largely unknown. Here, we found that auxin levels influence organ specification, and changes in auxin levels influence homeotic transformation between petals and stamens in rose (*Rosa hybrida*). The *PIN-FORMED-LIKES* (*PILS*) gene *RhPILS1* governs auxin levels in floral buds during floral organogenesis. *RhAUXIN RESPONSE FACTOR 18* (*RhARF18*), whose expression decreases with increasing auxin content, encodes a transcriptional repressor of the C-class gene *RhAGAMOUS* (*RhAG*), and controls stamen–petal organ specification in an auxin-dependent manner. Moreover, RhARF18 physically interacts with the histone deacetylase (HDA) RhHDA6. Silencing of *RhHDA6* increases H3K9/K14 acetylation levels at the site adjacent to the RhARF18-binding site in the *RhAG* promoter and reduces petal number, indicating that RhARF18 might recruit RhHDA6 to the *RhAG* promoter to reinforce the repression of *RhAG* transcription. We propose a model for how auxin homeostasis controls floral organ identity *via* regulating transcription of *RhAG*

## Teaser

The local distribution of auxin controls petal and stamen identity in the floral meristem of rose.

## Introduction

Rather than being single organs (like leaves or roots), flowers are composite structures composed of multiple organs arranged in an ordered pattern (Endress, 2010). A typical angiosperm flower consists of four types of organ: sepal, petal, stamen, and pistil. The number (merosity) and the arrangement (phyllotaxy) of floral organs vary in different species. Floral organs may differ in their position within a flower, in specific characteristics, or their identity, all of which are important criteria for homology (Theißen and Rümpler, 2018). Although different floral organs have different structures and functions, they all initiate from the floral meristem.

Flower patterning and determinacy is tightly controlled by a set of homeotic genes, whose functions are classified in the ABCE model (Coen and Meyerowitz, 1991; Weigel and Meyerowitz, 1994; Krizek and Fletcher, 2005; Theißen et al., 2016; Yan et al., 2016). All *ABCE* genes encode members of the MADS-box family, except *APETALA2* (*AP2*), which encodes a member of the AP2/ethylene-responsive element binding proteins (EREBPs) family. The formation of sepals is controlled by A-class genes *APETALA1* (*AP1*) and *AP2*, while the petals are coordinately controlled by A-class and B-class genes *AP3* and *PISTILLATA* (*PI*). Identity of stamens is determined by B-class genes and the C-class gene *AGAMOUS* (*AG*), and gynoecia are controlled by *AG*. The class E genes *SEPALLATA1–4* (*SEP1–SEP4*) encode functionally redundant proteins, which could interact with class A, B, C, and D proteins to form tetrameric complexes that specify each floral organ type.

Floral organ initiation and determination are also controlled by hormones, especially auxin. Auxin plays an indispensable role in floral organ initiation (Cheng and Zhao, 2007; Zhao, 2010; Yamaguchi et al., 2013). Local biosynthesis, metabolism, and transport of auxin are regulated coordinately to generate auxin maxima (Petrasek, 2006; Wisniewska, 2006; Křeček et al., 2009; Barbez et al., 2012; van Berkel et al., 2013; Adamowski and Friml, 2015; Brumos et al., 2018). Establishment of a local auxin maximum is required for the initiation of a flower primordium in *Arabidopsis thaliana* (Reinhardt et al., 2000; Benková et al., 2003; Heisler et al., 2005; Yamaguchi et al., 2013). In *A. thaliana*, a mutant of the auxin efflux carrier, *PIN-FORMED1* (*pin1-1*), produces no flowers in the apical meristem, and forms a pin-like stem (Gälweiler et al., 1998; Friml, 2003; Zhao, 2010). Overexpression of PIN-LIKES 3/5 (PILS3/5), the intracellular auxin carriers, in *A. thaliana* also results in deformation of flowers, inducing transitions of flower organs to flower buds, formation of extra gynoecia, and unfused carpels (Barbez et al., 2012). Similarly, mutation of the auxin-responsive transcription factor gene *MONOPTEROS*/*AUXIN-RESPONSIVE FACTOR5* (*MP*/*ARF5*), produces a pin-like stem (Hardtke and Berleth, 1998). Mutation of *ETTIN* (*ETT*)*/ARF3* leads to increased petal and sepal numbers and decreased stamen number, as well as defects in gynoecium (Sessions and Zambryski, 1995; Sessions et al., 1997). So far, however, whether and how auxin distribution and signaling are involved in controlling floral organ identity remains unknown.

Here, we found that changes in auxin levels induce homeotic transformation between petals and stamens in rose (*Rosa hybrida*). RhPILS1, a rose PILS1-type protein, governs auxin levels in floral buds during floral organogenesis. *RhARF18* is an auxin-regulated gene encoding a transcriptional repressor of *RhAG*, and thus plays a role in stamen–petal specification in an auxin-dependent manner. Moreover, we found that RhARF18 physically interacts with a histone deacetylase (HDA), RhHDA6. Therefore, we propose that RhARF18 might act as a subunit in a complex with RhHDA6 to control *RhAG* expression and petal–stamen fate *via* modifying histone acetylation level at the *RhAG* promoter.

## Results

### *RhPILS1* influences auxin homeostasis in floral meristem of rose

**Figure 1:**
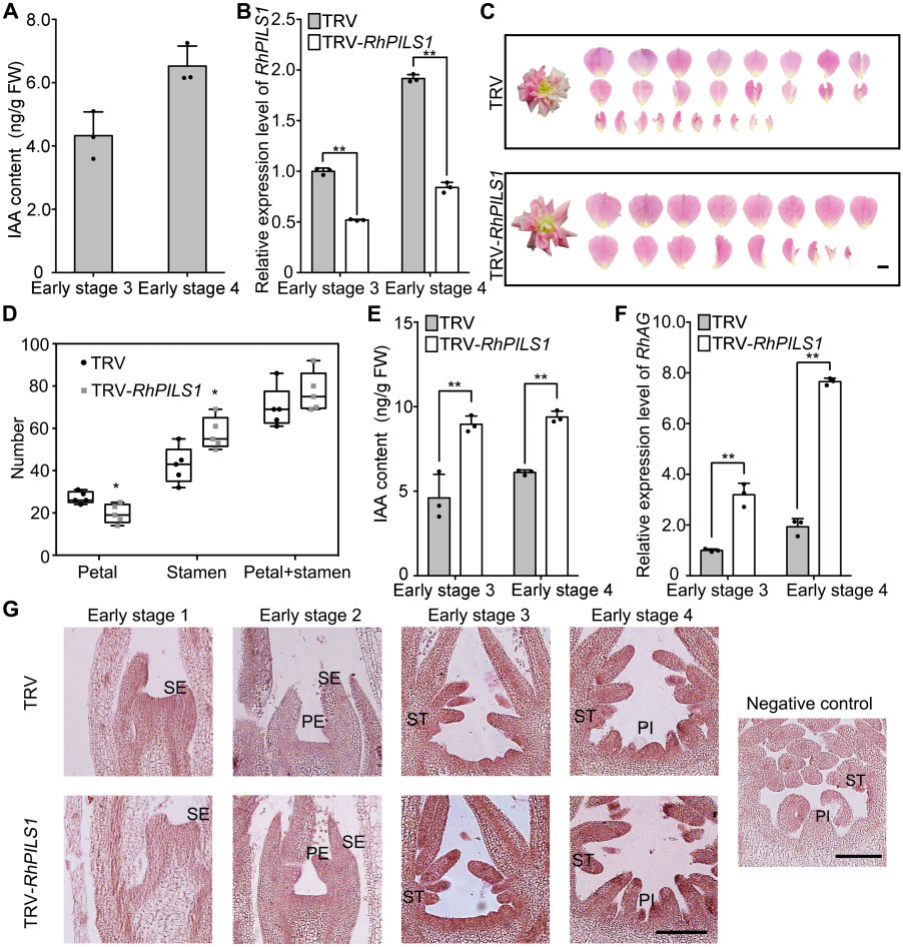
*RhPILS1* is involved in floral organ development *via* governing auxin level. A, Quantification of IAA levels in floral buds using UPLC–MS/MS. One biological sample consisted of a mixture of at least 12 or 8 floral buds at early stage 3 or 4, respectively. The mean values ± sd from three biological replicates (*n* = 3) are shown. B, Quantitative RT-PCR of *RhPILS1* in TRV control and *RhPILS1*-silenced floral buds. One biological sample consisted of a mixture of at least 12 or 8 floral buds at early stage 3 or 4, respectively. The mean values ± sd from three biological replicates (*n* = 3) are shown. Asterisks indicate statistically significant differences (two-sided Student's *t* test; **P < 0.01). *RhUBI2* was used as an internal control. C and D, Silencing of *RhPILS1* decreased petal numbers. Images of TRV and TRV-*RhPILS1* infected plants were taken 50–55 d after infiltration. The petals were digitally extracted for comparison. The number of petals, which included normal petals and petaloid stamens, and stamens were counted from 5 flowers (*n* = 5). The box itself contains the middle 50% of the data. The upper edge (hinge) of the box indicates the 75th percentile of the dataset while the lower hinge indicates the 25th percentile. The range of the middle two quartiles represents the inter-quartile range. The lines within the boxes indicate the median value of the data. Scale bar, 1 cm. Asterisks indicate statistically significant differences (two-sided Student's *t* test; *P < 0.05). E, Silencing of *RhPILS1* altered IAA level in floral buds. IAA levels were determined using UPLC–MS/MS in TRV control and *RhPILS1*-silenced floral buds. One biological sample consisted of a mixture of at least 12 or 8 floral buds at early stage 3 or 4, respectively. The mean values ± sd from three biological replicates (*n* = 3) are shown. Asterisks indicate statistically significant differences (two-sided Student's *t* test; **P < 0.01). (F and G, Silencing of *RhPILS1* influenced expression level of *RhAG*. Quantitative RT-PCR (F) and *in situ* hybridization (G) of *RhAG* were conducted in TRV control and in *RhPILS1*-silenced floral buds during floral organogenesis. The floral bud at early stage 4 probed with a sense probe was used as a negative control. At least 12 TRV and 12 TRV-*RhPILS1* plants were used for *in situ* hybridation. Scale bars, 200 μm. The mean values ± sd from three biological replicates (*n* = 3) are shown. Asterisks indicate statistically significant differences (two-sided Student's *t* test; **, *P* < 0.01). *RhUBI2* was used as an internal control. SE, sepal; PE, petal; ST, stamen; PI, pistil.

To test whether auxin homeostasis influences floral organ development in roses, we first monitored the auxin level in floral buds during rose flower development. The early development of floral buds is divided into four stages: sepal primordium initiation (early stage 1), petal primordium initiation (early stage 2), stamen primordium initiation (early stage 3), and pistil primordium initiation (early stage 4; Ma et al., 2015; Supplemental Figure S1A, upper panel). Immuno-gold localization analysis showed that IAA accumulated in the center region in early stages 3 and 4 (Supplemental Figure S2A). Ultra high-performance liquid chromatography–mass spectrometry/mass spectrometry (UPLC–MS/MS) assays further confirmed that levels of the major auxin indole-3-acetic acid (IAA) increased as flowers developed from early stage 3 to early stage 4 (Figure 1A), suggesting that auxin accumulation might be required for initiation of the floral primordium, especially stamen and pistil, in rose.

**Figure 2:**
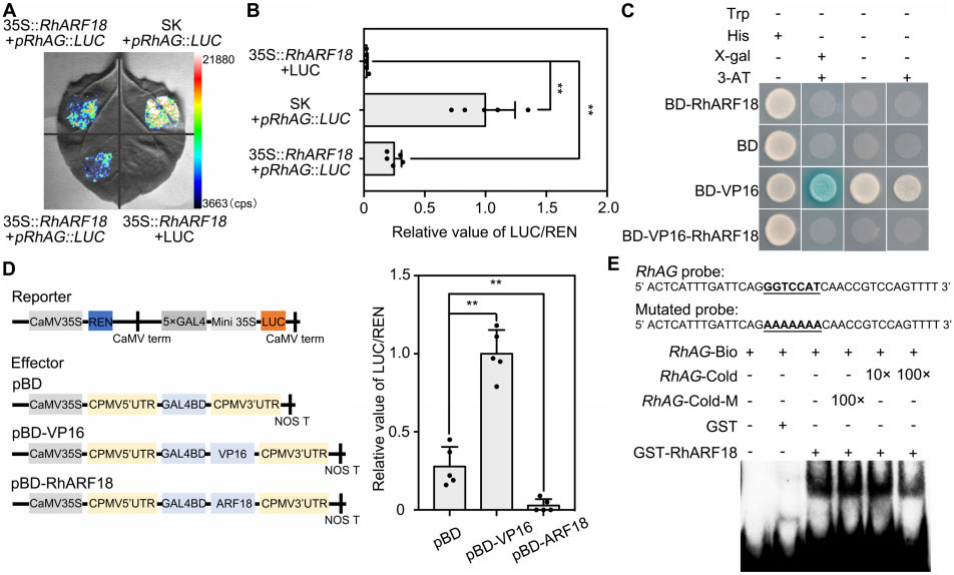
RhARF18 is a transcriptional repressor of *RhAG*. A, Transactivation of the *RhAG* promoter by RhARF18. The p*RhAG*::*LUC* construct was co-infiltrated with 35S::*RhARF18* or SK empty vector in *N. benthamiana* leaves. Co-infiltration of 35S::*RhARF18* plus LUC empty vector served as a negative control. The experiments were independently repeated three times. A representative image of an *N. benthamiana* leaf 72 h after infiltration is shown. B, Dual LUC reporter construct containing p*RhAG*::*LUC* and 35S::*REN* (*Renilla luciferase*) was co-infiltrated with 35S::*RhARF18* or SK empty vector in *N. benthamiana* leaves. Co-infiltration of 35S::*RhARF18* plus LUC empty vector served as a negative control. The experiments were performed independently twice, and similar results were obtained. The mean values ± sd are shown from five biological replicates (*n* = 5). Asterisks indicate statistically significant differences (two-sided Student's *t* test; **, *P* < 0.01). C, Analysis of the transcriptional activity of RhARF18 in yeast. The *RhARF18* and *RhARF18-VP16* sequences were cloned into the pBD-GAL4 Cam vector (GAL4BD). GAL4BD and GAL4BD-VP16 were used as a negative and positive control, respectively. The transformants were streaked on SD/-Trp, SD/-Trp-His, SD/-Trp-His+X-gal+3-AT, and SD/-Trp-His+3-AT plates, and incubated at 30°C for 3 d. The *β*-galactosidase activities were examined by X-gal staining. The experiments were performed independently twice, and similar results were obtained. D, Transcriptional repressor activity of RhARF18 in *N. benthamiana* leaves. (Left) schematic representation of Effectors and Reporter. The coding sequence with the stop codon was inserted into pBD effector driven by the 35S promoter. pBD-VP16 was used as a positive control. The mean values ± sd are shown from three biological replicates (*n* = 5). Asterisks indicate statistically significant differences (two-sided Student's *t* test; **P < 0.01). E, Analysis of RhARF18 binding to the promoter of *RhAG* in an EMSA system. The sequence of the region from -868 to -832 of the *RhAG* promoter was used as a probe (A of ATG marked for +1). As indicated, RhARF18-dependent mobility shifts were detected and competed by an unlabeled cold probe in a dose-dependent manner, but not by a mutated probe. The experiments were performed independently twice, and similar results were obtained.

To understand if regulation of auxin transport is involved in floral primordium initiation, we screened for auxin transporter genes against our Rose Flower Transcriptome Database (http://bioinfo.bti.cornell.edu/cgi-bin/rose_454/index.cgi). We identified two *PIN* and five *PIN-LIKES* (*PILS*) transcripts based on protein sequence alignment (Supplemental Table S1). Quantitative RT-PCR analysis showed that expressions of two *PINs* (*ARHL12440*, *ARHL05865*) and two *PIN-LIKES* genes (*ARHL01838*, *ARHL16576*) were relatively low during floral organ development (early stages 1–4), while *ARHL18978* and *ARHL25031* expressions remained at constant levels throughout the process (Supplemental Figure S1a, bottom panel).

Unlike other *PILS* genes, *ARHL22791* was highly expressed in floral organ development (early stages 1–4) and rapidly decreased when flower buds were evident, implying that ARHL22791 might be involved in early development of floral organs (Supplemental Figure S1a, bottom panel). Phylogenetic analysis indicated that the deduced amino acid sequence of ARHL22791 was close to that of the *PILS1*/*3*/*4* subclade in *A. thaliana* (Elena et al., 2012); hence, it was designated *RhPILS1* (Supplemental Figure S1B). Alignment of the deduced amino acid sequence indicated that RhPILS1 shared highly conserved domains with PILS1/3 proteins from other species, like woodland strawberry (*Fragaria vesca*), *Brassica rapa*, and *Populus euphratica* (Supplemental Figure S1C). Protein sequence analysis predicted 10 transmembrane domains and a short cytosolic fragment, consistent with previously reported topology of PILS in *A. thaliana* (Supplemental Figure S1D; Barbez et al., 2012). In addition, expression assays showed that *RhPILS1* was induced by auxin, like the typical auxin-inducible gene *GH3* (Hagen et al., 1984; Chapman and Estelle, 2009; Supplemental Figure S1E).

To test if *RhPILS1* plays a role in floral organ development in rose, we silenced *RhPILS1* in rose plants using virus-induced gene silencing (VIGS) technology with *Tobacco rattle virus* (TRV). Considering expression of *RhPILS1* was increasing as floral buds developed, we detected the expression of *RhPILS1* in floral buds at early stages 3 and 4. Quantitative RT-PCR assays demonstrated that expression of *RhPILS1* was significantly reduced in TRV-*RhPILS1* lines (Figure 1B). Compared to TRV-only controls, silencing of *RhPILS1* resulted in flowers with fewer layers of petals (Figure 1C). The petal number of *RhPILS1*-silenced plants was significantly less than that of TRV controls, while stamen number was significantly higher (Figure 1D). Meanwhile, the total numbers of petals and stamens in *RhPILS1*-silenced plants were similar to those of TRV plants (Figure 1D), suggesting that *RhPILS1* influences homeotic conversion between petals and stamens in rose flowers.

In *A. thaliana*, PILS has been reported to function as an auxin carrier to regulate intracellular auxin homeostasis. *PILS*-overexpressing *A. thaliana* plants show homeotic transformation of floral organs into new floral buds, triplication of the gynoecium, or unfused carpels (Barbez et al., 2012). We monitored auxin levels and distribution in floral buds of *RhPILS1*-silenced plants to explore whether silencing of *RhPILS1* caused petal and stamen conversion due to altered IAA level and distribution. UPLC–MS/MS assays showed that silencing of *RhPILS1* significantly elevated free IAA level in floral buds during early stages 3 and 4 (Figure 1E). Immuno-gold localization analysis also supported that silencing of *RhPILS1* enhanced IAA accumulation in stamen and pistil primordia (Supplemental Figure S2B).

Previous studies have reported that expression (levels and specific location) of *AGAMOUS* (*AG*), a C-class homeotic gene, are involved in controlling the floral organ identity of petals and stamens. In *A. thaliana*, the knockout *ag* mutant lacks normal pistils and stamens, and exhibits double flowers resulting from homeotic transformation of stamens to petals (Yanofsky et al., 1990). Our previous work also showed that knockdown of *RhAG* resulted in increased petal numbers and decreased stamen numbers in rose (Ma et al., 2015). Therefore, we speculate that silencing of *RhPILS1* might influence accumulation of *RhAG* transcripts in floral buds. Quantitative RT-PCR analysis confirmed that the expression level of *RhAG* in *RhPILS1*-silenced floral buds was significantly higher than in TRV controls in floral buds during early stages 3 and 4 (Figure 1F). *In situ* hybridization assays showed that *RhAG* transcripts accumulated to a higher level in the center of the floral meristem (early stage 3), as well as stamen and pistil primordia (early stage 4), in *RhPILS1*-silenced flowers than in TRV controls (Figure 1G). These results suggest that *RhPILS1* might affect petal–stamen specification *via* governing the distribution of endogenous auxin in flower primordia.

### RhARF18 directly represses transcription of *RhAG*

Given that silencing of *RhPILS1* influences auxin homeostasis and *RhAG* accumulation, auxin signaling might be involved in the regulation of *RhAG* expression, and thus controls petaloidy of rose stamens. We searched the putative *cis-*elements in the promoter region of *RhAG* using the PlantCARE program (http://bioinformatics.psb.ugent.be/webtools/plantcare/html/). We found an AuxRR-core *cis*-element GGTCCAT at −853 to −847 bp upstream to the ATG of *RhAG* (Supplemental Figure S3A). The AuxRR *cis*-element is different from canonical auxin response elements (TGTCCC/GG/TC/AC; Lai et al., 2019; Lieberman-Lazarovich et al., 2019), and it was identified in the promoter of an auxin-responsive *cellulose synthase catalytic subunit 4* (*CesA4*) gene in *Gossypium hirsutum* (Kim et al., 2011). Next, we searched for *ARFs* in the rose genome and checked their expression during flower development using our Rose Transcriptome Database (http://bioinfo.bti.cornell.edu/cgi-bin/rose_454/index.cgi). We identified 12 *ARFs* and found that 8 of them were detectable by RNA sequencing in floral buds (Pei et al., 2013a, 2013b; Supplemental Table S2).

**Figure 3:**
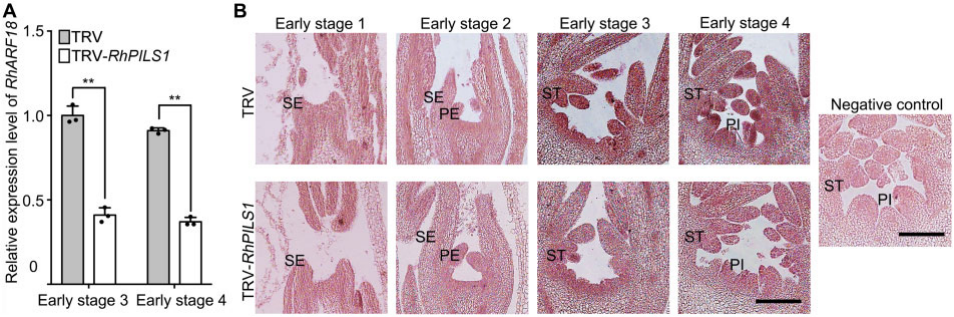
Silencing of *RhPILS1* represses accumulation of *RhARF18* transcripts in rose floral buds. A, Quantitative RT-PCR of *RhARF18* in TRV control and *RhPILS1*-silenced floral buds. One biological sample consisted of a mixture of at least 12 or 8 floral buds at early stage 3 or 4, respectively. The mean values ± sd from three biological replicates (*n* = 3) are shown. Asterisks indicate statistically significant differences (two-sided Student's *t* test; **P < 0.01). *RhUBI2* was used as an internal control. B, *In situ* hybridization of *RhARF18* in TRV control (upper) and *RhPILS1*-silenced flower buds (lower panel). The floral bud at early stage 4 probed with a sense probe was used as a negative control. At least 12 TRV and 12 TRV-*RhPILS1* plants were used. Scale bars, 200 μm. SE, sepal; PE, petal; ST, stamen; PI, pistil.

We then conducted a transactivation assay to identify the ARFs that are able to regulate expression of the firefly luciferase (*LUC*) gene driven by the *RhAG* promoter in *Nicotiana benthamiana* leaves. Each *RhARF* ORF was driven by the 35S promoter and was co-infiltrated with p*RhAG*::*LUC* reporter into *N. benthamiana* leaves, while co-infiltration of empty SK plasmid and p*RhAG*::*LUC* served as a control. We found that ARHL06399 strongly repressed activity of the *LUC* reporter, while most of the tested *ARFs* barely influenced it (Supplemental Figure S3B). Therefore, we chose ARHL03699 for further analysis.

According to the annotation of the rose genome (Raymond et al., 2018), ARHL06399 was designated as *RhARF18*. Phylogenetic analysis showed that *RhARF18* is close to *A. thaliana ARF16* (Supplemental Figure S4A). Protein sequence analysis predicted a B3 DNA-binding domain, an Auxin Response Factor domain, and an AUX/IAA family domain in RhARF18, similar to ARF16 (Supplemental Figure S4, B and C). Quantitative RT-PCR showed that expression levels of RhARF18 dropped in floral buds from early stages 1 to 4 (Supplemental Figure S4D). Fluorescence co-localization showed that RhARF18 protein was localized in the nucleus, indicating that RhARF18 could function as a transcriptional regulator (Supplemental Figure S4E).

**Figure 4:**
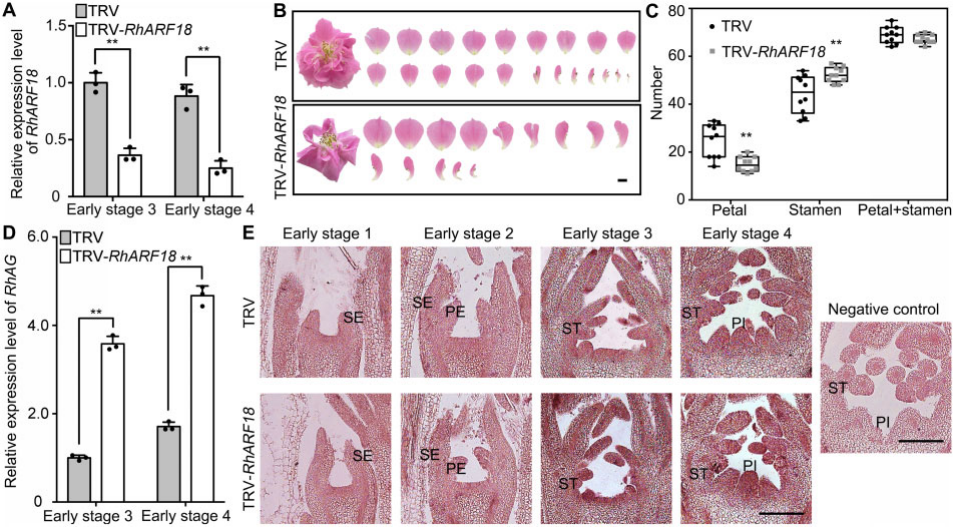
Silencing of *RhARF18* alters stamen petaloidy and expression of *RhAG* in rose floral buds. A, Quantitative RT-PCR of *RhARF18* in TRV control and *RhARF18*-silenced floral buds. One biological sample consisted of a mixture of at least 12 or 8 floral buds at early stage 3 or 4, respectively. The mean values ± sd from three biological replicates (*n* = 3) are shown. Asterisks indicate statistically significant differences (two-sided Student's *t* test; **P < 0.01). *RhUBI2* was used as an internal control. B and C, Silencing of *RhARF18* reduced petal number. Images of TRV and TRV-*RhARF18* infected plants were taken 50–55 d after infiltration. The petals were digitally extracted for comparison. The number of petals (which included normal petals and petaloid stamens) and stamens were counted from 10 flowers (*n* = 10). Images representative of three independent experiments are shown. The box itself contains the middle 50% of the data. The upper edge (hinge) of the box indicates the 75th percentile of the dataset while the lower hinge indicates the 25th percentile. The range of the middle two quartiles represents the inter-quartile range. The lines within the boxes indicate the median value of the data. Scale bar, 1 cm. Asterisks indicate statistically significant differences (two-sided Student's *t* test; **P < 0.01). D, Quantitative RT-PCR of *RhAG* in TRV control and *RhARF18*-silenced floral buds. One biological sample consisted of a mixture of at least 12 or 8 floral buds at early stage 3 or 4, respectively. The mean values ± sd from three biological replicates (*n* = 3) are shown. Asterisks indicate statistically significant differences (two-sided Student's *t* test; **P < 0.01). *RhUBI2* was used as an internal control. E, *In situ* hybridization of *RhAG* in TRV control (upper) and in *RhARF18*-silenced flower buds (lower) during floral organogenesis. The floral bud at early stage 4 probed with a sense probe was used as a negative control. Images representative of three independent experiments are shown. For each experiment, at least 13 TRV and 13 TRV-*RhARF18* plants were used. Scale bars, 200 μm. SE, sepal; PE, petal; ST, stamen; PI, pistil.

A dual-LUC reporter assay confirmed that RhARF18 significantly repressed the transactivation of the *RhAG* promoter (Figure 2, A and B). Once *RhARF18* was fused to the VP16 activator, the transactivation activity of VP16 was totally repressed in yeast (Figure 2C). Moreover, pBD-RhARF18 significantly repressed expression of the LUC reporter in comparison to the effect of pBD alone, suggesting that RhARF18 is a transcriptional repressor (Figure 2D). To determine whether RhARF18 directly binds to the GGTCCAT element in the *RhAG* promoter, we conducted an electrophoretic mobility shift assay (EMSA). RhARF18 was expressed as a GST fusion protein in *Escherichia coli* strain Rosetta, and recombinant GST-RhARF18 proteins were induced by adding IPTG. Shifted bands were detected in the presence of recombinant GST-RhARF18 proteins and biotin-labeled probes containing the AuxRR *cis*-element. The intensity of the shifted bands was decreased by increasing concentrations of cold competitor probe (nonbiotin-labeled), but was not affected by adding mutated cold probe (Figure 2E), further supporting that this AuxRR *cis*-element can be specifically recognized by RhARF18. These results indicated that RhARF18 was able to directly bind the AuxRR *cis*-element in the *RhAG* promoter and suppress its transcription activity.

We next monitored the expression pattern of *RhARF18* in *RhPILS1*-silenced floral buds during the floral organ development process using RT-qPCR and *in situ* hybridization. Quantitative RT-PCR analysis demonstrated that silencing of *RhPILS1* significantly reduced expression levels of *RhARF18* in floral buds during early stages 3 and 4 (Figure 3A). In addition, *in situ* hybridization showed that transcript levels of *RhARF18* in stamen and pistil primordia were weaker in *RhPILS1*-silenced flower buds than in TRV controls (Figure 3B), indicating that *RhARF18* level might be negatively associated with auxin content.

Notably, silencing of *RhARF18* led to a flower phenotype similar to that of *RhPILS1*-silenced rose plants, including substantially decreased petal number and increased stamen number compared to TRV controls (Figure 4A–C). The total number of petals and stamens was not altered between *RhARF18*-silenced and TRV plants, suggesting that RhARF18 might be involved in specification of stamens and petals in roses (Figure 4C). Consistent with this idea, RT-qPCR demonstrated that the expression level of *RhAG* was significantly increased by silencing of *RhARF18* in floral buds during early stages 3 and 4 (Figure 4D). *In situ* hybridization also showed that *RhAG* mRNA accumulated to a higher level in *RhARF18*-silenced floral buds than in TRV controls, especially in the stamen and pistil primordia (Figure 4E). These results indicate that RhARF18 is involved in specification of stamens and petals via governing expression of *RhAG*.

### RhARF18 recruits RhHDA6 to the promoter of *RhAG*

To understand how RhARF18 functions as a transcriptional repressor in rose flower, we used a yeast two-hybrid (Y2H) system to screen a cDNA library from rose floral buds for potential RhARF18 interactors in . The screen yielded 52 potential interacting proteins, of which 11 candidates were identified independently at least twice (Supplemental Table S3). Among the interacting proteins, we noticed a HDA, ARHL09507. Phylogenetic analysis showed that ARHL09507 was close to HDA6 from *A. thaliana* (Supplemental Figure S5A). Conserved domain analysis predicted the HDA domain (36–327 aa; http://pfam.xfam.org), and thus we named the ARHL09507 protein RhHDA6 (Supplemental Figure S5B). Expression of *RhHDA6* remained relatively constant during floral organogenesis (early stages 1–4; Supplemental Figure S5C). Subcellular localization demonstrated that RhHDA6 localized in the nucleus (Supplemental Figure S5D).

**Figure 5:**
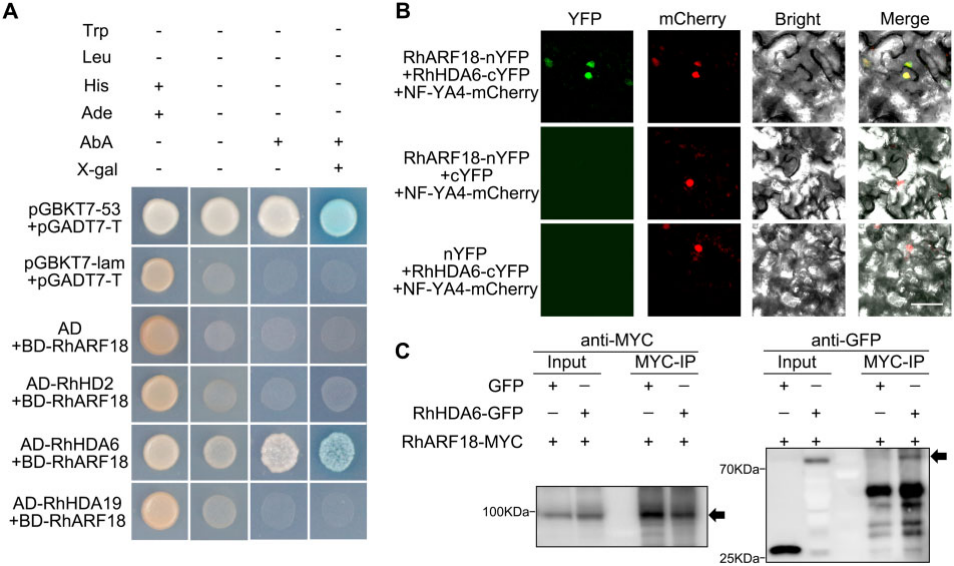
RhARF18 interacts with HDA RhHDA6. A, RhARF18 and RhHDA6 interaction in yeast. AD is empty vector of pGADT7. Positive control is pGBKT7-53 + pGADT7-T, while negative control is pGBKT7-lam + pGADT7-T. B, Interaction of RhARF18 and RhHDA6 in a bimolecular fluorescence complementation assay. *RhARF18-nYFP* was co-infiltrated with *RhHDA6-cYFP* in *N. benthamiana* leaves. Infiltrated leaves were visualized by confocal microscopy 3 d after infiltration. *RhARF18-nYFP* with *cYFP* and *nYFP* with *RhHDA6-cYFP* were used as negative controls. *NF-YA4-mCherry* was co-infiltrated as a nuclear marker. The experiment was performed independently three times, and representative results are shown. Scale bar, 50 μm. C, Co-immunoprecipitation assays of RhARF18 and RhHDA6 in *N. benthamiana* leaves. The RhARF18-MYC was co-infiltrated with RhHDA6-GFP in *N. benthamiana* leaves. The total proteins were extracted 3 d after infiltration and the supernatant with soluble proteins was incubated with anti-MYC antibody. The precipitates were analyzed by western blotting by using anti-MYC and anti-GFP antibodies.

Y2H assays showed that RhARF18 interacted with RhHDA6 *in vivo*, but not with HDA19 or HD2 (Figure 5A). Both HDA19 and HDA6 are members of Class I of the *RPD3*/*HDA1* family, while HD2 is a plant-specific HDA (Liu et al., 2014); our results suggested that RhARF18 interacted specifically with RhHDA6. A bimolecular fluorescence complementation (BiFC) assay showed that RhARF18 interacted with RhHDA6 in the nucleus in *N. benthamiana* leaves (Figure 5B). To further confirm the interaction between RhARF18 and RhHDA6, we performed immunoprecipitation of RhARF18 and RhHDA6. *RhARF18-MYC* and *RhHDA6-GFP* were co-expressed in *N. benthamiana* leaves, and the possible protein complexes were precipitated using anti-MYC antibody and then probed with anti-GFP antibody. The results showed that RhHDA6 was co-immunoprecipitated with RhARF18 (Figure 5C), confirming that RhARF18 interacts with RhHDA6 *in planta*.

**Figure 6:**
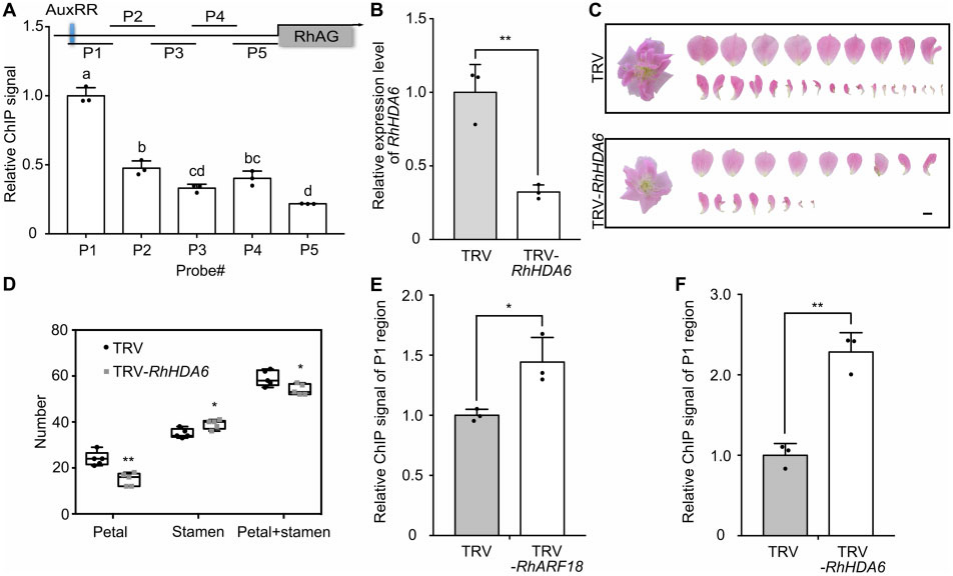
*RhHDA6* regulates histone acetylation level in the promoter of *RhAG* and is involved in floral organ development. A, Schematic diagram of the RhARF18-recognized site (upper) and relative H3K9/K14Ac level in the *RhAG* promoter (bottom). The mean value ± sd are shown from three biological replicates (*n* = 3). Different letters above each bar indicate signiﬁcant differences according to one-way ANOVA with Tukey's multiple comparisons test (*P* < 0.05). The blue box represents AuxRR *cis*-element. B, Quantitative RT-PCR of *RhHDA6* in TRV and TRV-*RhHDA6* floral buds. At least eight floral buds were mixed as one biological sample of early stage 4. The mean values ± sd from three biological replicates (*n* = 3) are shown. Asterisks indicate statistically significant differences (two-sided Student's *t* test; **P < 0.01). *RhUBI2* was used as an internal control. C and D, Silencing of *RhHDA6* decreased petal number. Images of TRV and TRV-*RhHDA6* infected plants were taken 50–55 d after infiltration. The petals were digitally extracted for comparison. The number of petals and stamens were counted from five flowers (*n* = 5). Images representative of three independent experiments are shown. The box itself contains the middle 50% of the data. The upper edge (hinge) of the box indicates the 75th percentile of the dataset while the lower hinge indicates the 25th percentile. The range of the middle two quartiles represents the inter-quartile range. The lines within the boxes indicate the median value of the data. Scale bar, 1 cm. Asterisks indicate statistically significant differences (two-sided Student's *t* test; *P < 0.05; **P < 0.01). E and F, Relative levels of H3K9/K14Ac in the promoter of *RhAG* in *RhARF18*-silenced (E) and *RhHDA6*-silenced (F) rose plants. Measurement of H3Ac enrichment on the P1 region promoter of *RhAG* assessed by ChIP. Asterisks indicate statistically significant differences (two-sided Student's *t* test; *P < 0.05; **P < 0.01).

Previous reports have demonstrated that HDA6 controls the H3K9/K14 acetylation level in plants (Yu et al., 2011; Luo et al., 2012). Therefore, we monitored the H3 acetylation level of the *RhAG* promoter in rose floral buds at early stage 4. Interestingly, a relatively high level of H3K9/K14 acetylation was detected in a region adjacent to the RhARF18-recognized site (P1, −861 to −644; Supplemental Table S4) in the *RhAG* promoter (Figure 6A).

We silenced *RhHDA6* using the VIGS approach to confirm the involvement of *RhHDA6* in floral organogenesis. To avoid cross-silencing, we chose a *RhHDA6*-specific fragment to construct the TRV-*RhHDA6* plasmid, and measured expression levels of *RhHDA6* and other members of the *RPD3*/*HDA1* family in TRV-*RhHDA6*-infected floral buds (Supplemental Table S5). Quantitative RT-PCR showed that specifically *RhHDA6* was silenced (Figure 6B;Supplemental Figure S6). Interestingly, silencing of *RhHDA6* significantly decreased petal number but increased stamen number (Figure 6, C and D), suggesting that *RhHDA6* is involved in regulation of petal and stamen development. Furthermore, silencing of either *RhHDA6* or *RhARF18* significantly elevated the H3K9/K14 acetylation level in the P1 region (Figure 6, E and F), implying that RhARF18 might recruit RhHDA6 to the *RhAG* promoter to modify histone acetylation level of the latter.

Taken together, our findings suggest a model for auxin homeostasis in the petal–stamen homeotic transition in rose. RhPILS1 regulates free auxin level and distribution in the floral meristem. As an auxin-responsive repressor, RhARF18 governs petal and stamen development by directly suppressing transcription of the C-class gene *RhAG*. Moreover, RhARF18 might be able to recruit the HDA RhHDA6 to the *RhAG* promoter to reinforce the transcription suppression. Elevated auxin levels would suppress expression of *RhARF18*, and thus release expression of *RhAG*, leading to the homeotic transition from petals to stamens (Supplemental Figure S7).

## Discussion

In plants, auxin regulates cell division, expansion, and differentiation in both shoot and root meristems, and thus auxin homeostasis plays a vital role in organogenesis, leading to proper plant architecture. Local biosynthesis and polar transport action coordinately establish the morphogenic auxin maxima that trigger primordium initiation (Zhao, 2010; Brumos et al., 2018). The auxin efflux mutant *pin1* generates a flower with wide petals that lack stamens and ovules at the top of inflorescence axes (Okada et al., 1991), while *pin6-2* had petals that failed to expand, smaller nectaries, and lacked short stamens (Bender et al., 2013).

Besides the classical PINs charged with intercellular transport of auxin, a PILS family of auxin carriers has been discovered to control intracellular auxin homeostasis (Barbez et al., 2012; Adamowski and Friml, 2015). In *A. thaliana*, PILS proteins localize in the endoplasmic reticulum (ER) and control auxin availability for nuclear auxin signaling (Barbez et al., 2012). Ectopic expression of *PILS* genes, like *PILS3*, produces severe defects in flowers, such as homeotic transformation of flower organs into new flower buds, triplication of the gynoecium, or unfused carpels. In our study, we found that silencing of *RhPILS1*, a RhPILS1-type protein in rose, led to homeotic conversion between petals and stamens in rose. Compared to Col-0, free IAA level is significantly higher in *pils2* and *pils5* mutants (Barbez et al., 2012). Similarly, IAA quantifications and immuno-gold assay showed that free IAA was elevated in *RhPILS1*-silenced rose floral buds, especially in stamen and pistil primordia. Moreover, the increased auxin caused increased numbers of stamens but decreased numbers of petals in *RhPILS1*-silenced rose floral buds, indicating that auxin level and distribution governs proper floral organ development. Notably, a previous study reported that in *A. thaliana*, new floral primordia will initiate in SAM at the region with the highest concentration of auxin (Heisler et al., 2005). A boundary gene, *SUPERMAN* (*SUP*), is able to control floral organogenesis and floral meristem determinacy *via* governing local auxin distribution and signaling in *A. thaliana*. SUP protein can directly repress expression of the auxin biosynthesis genes *YUCCA1*/*4* (*YUC1*/*4*). The *sup* mutant exhibits an enhanced auxin level at the boundary between whorls 3 and 4 due to depression of *YUC1*/*4*, and exhibits an increased number of stamen (Xu et al., 2018). In addition to *YUC1*/*4*, SUP seems to regulate expression of *PIN3*/*4*, and whether SUP regulates the expression of *PILS* genes would be an interesting question. On the other hand, whether and how auxin biosynthesis is regulated in rose floral buds need to be clarified in the future.

Elevated IAA levels suppressed expression of an AUXIN RESPONSE FACTOR gene, *RhARF18*, in rose. Usually, ARF transcription factors are activated through the auxin-dependent degradation of the Aux/IAA repressors (Chapman and Estelle, 2009). Although most ARFs are auxin activation factors (AAFs), several ARFs could be downregulated by auxin. In *Medicago truncatula*, *MtARF18*, a homolog of *RhARF18*, is also repressed by auxin in both shoots and roots (Shen et al., 2015). How auxin represses the expression of *RhARF18* is yet to be clarified.

RhARF18 directly bound to the AuxRR *cis*-element of the *RhAG* promoter and transcriptionally suppressed expression of *RhAG*. Silencing of *RhARF18* upregulated expression of *RhAG* and resulted in homeotic transformation from petals to stamens. In *A. thaliana*, *AG* expression is restricted by AP2 to the inner two floral whorls (Drews et al., 1991). AINTEGUMENTA (ANT) acts redundantly with AP2 to repress *AG* in the second whorl (Krizek et al., 2000). LEUNIG (LUG), a glutamine-rich protein with seven WD repeats, acts as a transcriptional regulator to govern the expression of *AG* expression in the outer two whorls of a flower. In *lug* and *ap2* mutants, ectopically expressed *AG* in the outer two whorls leads to homeotic transformation from sepals to stamens or carpels, and petals that are either staminoid or absent (Drews et al., 1991; Liu and Meyerowitz, 1995). In rose, the expression region of *RhAG* is restricted toward the center of floral buds in cultivars with double flowers, while it is wider in cultivars with single flowers (Dubois et al., 2010). Low temperature could induce homeotic transformation from petals to stamens *via* attenuating expression of *RhAG* (Ma et al., 2015). Whether auxin mediates the effects of low temperature on floral organ development and how RhARF18 coordinates with other upstream regulators to control expression of *RhAG* would be interesting topics to study in the future.

Because RhARF18 physically interacts with RhHDA6 and silencing of *RhHDA6* increases H3K9/K14 acetylation level at the site adjacent to the RhARF18-binding site in the *RhAG* promoter, we speculate that RhARF18 might recruit RhHDA6 to the *RhAG* promoter to reinforce the transcription repression of *RhAG* through histone deacetylation. In *A. thaliana*, auxin-induced MP/ARF5 recruits BRM-containing or SYD-containing chromatin remodelers to increase chromatin accessibility of the genes that are crucial for floral organ initiation (Wu et al., 2015; Chung et al., 2019). Another ARF, ETTIN (ETT)/ARF3, also plays a pivotal role in floral organ development, and the *arf3/ett* mutant exhibits increased petal number, decreased stamen number, decreased anther formation, and aberrant gynoecium morphogenesis (Sessions et al., 1997). A recent unexpected report demonstrates that ETT/ARF3 protein interacts with a TOPLESS/TOPLESS-RELATED (TPL/TPR)–HDA19 complex to repress transcription at ETT/ARF3 target loci *via* H3K27 deacetylation. Under high auxin conditions, ETT/ARF3 protein directly binds auxin molecules, resulting in disassociation of ETT/ARF3 from the complex, releasing expression of ETT/ARF3 targets (Kuhn et al., 2020). Similarly, AP2 interacts with the TPL/TPR–HDA19 complex to repress expression of *AG* in the outer two whorls (Krogan et al., 2012). Although HDA19 is close to HDA6, our results showed that RhARF18 specifically interacts with RhHDA6 but not RhHDA19. Whether RhARF18 could interact RhHDA19 indirectly, for example through TPL/TPR proteins, needs to be clarified. Notably, AP2-TPL/TPR-HDA19 targets the second intron of *AG* in *A. thaliana*, while RhARF18–RhHDA6 targets the upstream promoter of *RhAG*, indicating that *AG* is extensively regulated by multiple pathways.

Intriguingly, *CRABS CLAW* (*CRC*), a direct target of AG, is recently reported to directly repress transcription of a plasma membrane-localized protein *TORNADO2* (*TRN2*) in a flower-specific manner. Plants with mutation of *TRN2* display reduced IAA transport compared to wild type, indicating that AG-CRC-TRN2 is involved in auxin homeostasis in floral meristem and is crucial to floral organ development (Yamaguchi et al., 2017). Moreover, both AG and CRC are able to bind to the promoter of *YUCCA4* (*YUC4*), an auxin biosynthetic gene, suggesting that AG plays an important role in auxin biosynthesis as well (Yamaguchi et al., 2018). Therefore, auxin homeostasis (biosynthesis and conjugation) and *AG* level might form a regulatory loop to precisely govern floral organ development.

## Conclusions

In this study, we have demonstrated that the auxin-responsive gene *RhARF18* controls petal–stamen specification by directly suppressing expression of the C-class gene *RhAG*. RhARF18 recruits HDA RhHDA6 to the *RhAG* promoter to reinforce the transcription repression *via* histone deacetylation. This work identifies a regulatory pathway by which auxin homeostasis governs floral organ development in rose.

## Materials and methods

### Plant materials and growth conditions

*Rosa hybrida* cv “Samantha” and *N. benthamiana* plants were grown as described previously (Zhang et al., 2019). Rose stems with at least one node were cut and used as explants, and cultured on Murashige and Skoog (MS) medium (Duchefa Biochemie, Haarlem, Netherlands) supplemented with 1.0 mg/L 6-benzyl aminopurine (6-BA), 3 mg/L Gibberellin acid 3 (GA_3_), and 0.05 mg/L 1-naphthaleneacetic acid (NAA) for 30 d at 22 ± 1°C, under a 16-h light/8-h dark photoperiod. The shoots were then transferred to half-strength MS medium supplemented with 0.1 mg/L NAA for 25 d for rooting. Then the plants were transferred to pots containing peat moss:vermiculite (1:1) and were grown at 22 ± 1°C with a relative humidity of ∼60% and 16 h light/8 h dark photoperiod. *Nicotiana benthamiana* were planted in pots in a growth chamber with the same growth conditions.

### Virus-induced gene silencing

VIGS was performed as previously described (Zhang et al., 2019). A gene-specific fragment of *RhPILS1* (311 bp in length), *RhARF18* (346 bp in length), and *RhHDA6* (235 bp in length) were used to construct the vectors pTRV2-*RhPILS1*, pTRV2-*RhARF18*, and pTRV2-*RhHDA6*, respectively. pTRV1, pTRV2 and pTRV2-*RhPILS1*, pTRV2-*RhARF18*, and pTRV2-*RhHDA6* were transformed into *Agrobacterium tumefaciens* strain GV3101. *Agrobacterium tumefaciens* strain GV3101 carrying the constructed vector was grown at 28°C in Luria–Bertani medium supplemented with 20 mM acetosyringone, 50 mg/L rifampicin and 50 mg/L kanamycin, and the *A. tumefaciens* culture was shaken on a rocking platform at 200 rpm for approximately 18 h. *Agrobacterium tumefaciens* cells were harvested by centrifugation at 4°C and re-suspended in the infiltration buffer (10 mM MgCl_2_, 200 mM acetosyringone and 10 mM 2-(N-Morpholino)-ethanesulfonic acid, pH 5.6; final absorbance A600 = 1.5). A mixture of *A. tumefaciens* cultures containing pTRV1 and pTRV2-*RhPILS1*/*RhARF18*/*RhHDA6* in a ratio of 1:1 (v/v), or with pTRV1 and pTRV2 (the negative control), was placed in the dark at room temperature for 4 h before inoculation. Rose plantlets were submerged in infiltration buffer containing pTRV1 and pTRV2-*RhPILS1*/*RhARF18*/*RhHDA6* (A600 = 0.8) and exposed to a vacuum of −25 kPa twice, each for 60 s. The infiltrated plants were briefly washed with distilled water and planted in pots for subsequent analysis. The phenotype of rose plants was recorded 50–55 d after infiltration. The buds of rose plants were monitored and were cut at various time points during floral organogenesis. The primers used in VIGS are listed in Supplemental Table S6.

### Quantification of endogenous auxins

About 50 mg of floral buds were harvested from rose plants for measurement of auxin content. Phytohormones were extracted from 50 mg of ground floral buds using cold methanol containing internal standards. Measurement of free IAA was carried out using a UPLC–MS/MS platform consisted of an Acquity UPLC (Waters Corp.) and a Q-Exactive (Thermo Scientific) mass spectrometer. Three biological replicates were performed for each sample.

### Immuno-gold localization of IAA

The immuno-gold localization of endogenous IAA was performed as described previously (Liu et al., 2018; Shen et al., 2019). Excised floral buds were fixed in 2% (w/v) N-(3-Dimethylaminopropyl)-N′-ethylcarbodiimide hydrochloride (Sigma, St Louis, USA) by vacuum infiltration for 2 h and then transferred into 4% (w/v) paraformaldehyde and 1% (v/v) glutaraldehyde for 7 h at 4°C. The sections were incubated with Anti-Rabbit IgG–Gold antibody (1:50; Sigma, St Louis, USA) at 37°C for 1 h and stained with developing solution.

### Phylogenetic analyses

Phylogenetic analyses were performed as described previously (Elena et al., 2012). Only the conserved domains were used and all positions with <80% site coverage were eliminated. The evolutionary history was inferred by using the Maximum Likelihood method based on models which are analyzed and recommended in MEGA software (version X). The phylogenetic tree was constructed based on LG + G model for analysis of PIN and PILS amino acid sequences, WAG + G model for analysis of ARF amino acid sequences and LG + G model for analysis of HDA amino acid sequences.

### *In situ* hybridization

*In situ* hybridization was performed as described previously (Ma et al., 2008, 2015). The floral buds were fixed in 4% (w/v) paraformaldehyde by vacuum infiltration for 2 h and then incubated overnight at 4°C. The paraffin sections were processed and incubated with a DIG-labeled RNA probe overnight at 50°C. The *RhAG* and *RhARF18* probes were 417-bp and 346-bp linearized fragments, respectively, from the unique region of the corresponding coding sequence. The primers used in *in situ* hybridization are listed in Supplemental Table S6.

### RNA extraction and quantitative RT-PCR

Whole flower buds were harvested and frozen in liquid nitrogen. Total RNA was extracted by the hot borate method as described previously (Ma et al., 2015). The cDNA templates for RT-qPCR were made with a HiScript II reverse transcriptase kit (Vazyme, Nanjing, China). StepOne Real-Time PCR System (Applied Biosystems, Carlsbad, USA) and the KAPA SYBR FAST Universal RT-qPCR kit (Kapa Biosystems, Boston, USA) were used in RT-qPCR reactions. *RhACT5* or *RhUBI2* was used as an internal control. The primers used in RT-qPCR are listed in Supplemental Table S6.

### Transactivation assays

For the transactivation assay in yeast, coding sequence (CDS) of *RhARF18*, *VP16*, and *RhARF18*-*VP16* were constructed into pGBKT7 and then transformed into yeast strain AH109. pBD-*VP16* was used as a positive control and the empty vector pGBKT7 was used as a negative control. The transformants were then spotted onto SD/-Trp, SD/-Trp-His, SD/-Trp-His + 3-amino-1,2,4-triazole and SD/-Trp-His+X-gal + 3-amino-1,2,4-triazole.

Transcription activity analysis of RhARF18 in *N. benthamiana* was performed as described previously (Han et al., 2016). Briefly, the coding sequence of *RhARF18* was inserted into the constructed pBD vector driven by the 35S promoter as an effector. The effector was introduced into *Agrobacterium* GV3101 and then co-infiltrated into *N. benthamiana* leaves with a double-reporter vector, which harbors a GAL4-LUC and an internal control REN driven by the 35S promoter. pBD-*VP16* was used as a positive control and the empty vector pBD-empty was used as a negative control. The primers used in transactivation assays are listed in Supplemental Table S6.

### Electrophoresis mobility shift assay

EMSA was performed as described previously (Pei et al., 2013b). The *RhARF18* CDS was fused in-frame to GST and expressed in *E. coli* strain Rosetta. The fused protein was induced by adding isopropylthio-b-galactoside (0.4 mM), and the cells were incubated at 160 rpm for 12 h at 16°C. The recombinant protein was purified using Glutathione Sepharose 4B (GE Healthcare, Pittsburgh, USA) according to the manufacturer's instructions. EMSAs were performed using Chemiluminescent Nucleic Acid Detection Module Kit (Thermo Fisher, Waltham, USA) according to the manufacturer's instructions. The primers used in EMSA are listed in Supplemental Table S6.

### Y2H assays

A Y2H system was used to screen for RhARF18 interaction proteins in a cDNA library from rose floral buds (mixed from early stages 1–4). The coding region sequence of *RhARF18* was constructed into pGBKT7 (BD) as bait. Alignment with the NCBI database revealed 52 potential interaction proteins, which are listed in Supplemental Table S3. The CDS of *RhHD2*, *RhHDA6*, and *RhHDA19* were constructed into pGADT7 (AD) as prey vectors. pGBKT7-53 and pGADT7-T were used as positive controls and pGBKT7-lam and pGADT7-T were used as negative controls. The bait and the prey vectors were co-transformed into yeast strain Y2H Gold. The transformants were then spotted onto SD/-Trp-Leu, SD/-Trp-Leu-His-Ade, SD/-Trp-Leu-His-Ade+Aureobasidin A and SD/-Trp-Leu-His-Ade+Aureobasidin A + X-gal. The primers used in Y2H assays are listed in Supplemental Table S6.

### Dual LUC assay for protein–DNA interactions in *N. benthamiana* leaves

The dual-LUC reporter assay was performed as described previously (Gao et al., 2019). The CDS of each *RhARF* was inserted into the pGreenII 0029 62-SK plasmid to construct effectors, and the promoter of *RhAG* was inserted into pGreenII 0800-LUC plasmid to construct the reporter. Effector, reporter and pSoup were co-transfected into *N. benthamiana* leaves (Hellens et al., 2000). For live LUC imaging, 1 mM luciferin was sprayed onto leaves 3 d after infiltration, and the plants were kept in dark for 5 min to quench the fluorescence. A low-light cooled CCD imaging apparatus (CHEMIPROHT 1300B/LND, 16 bits; Roper Scientific) was used to capture the LUC image at −110°C. LUC and REN activity were measured by the dual-LKC reporter assay reagents kit (Promega, Madison, USA) and a GloMax 20/20 luminometer (Promega) 3 d after infiltration. The primers used in LUC assays are listed in Supplemental Table S6.

### Bimolecular fluorescence complementation

For the BiFC assay, RhARF18 was fused with the N terminus of YFP (nYFP) and RhHDA6 was fused with the C terminus of YFP (cYFP). *RhARF18-nYFP* and *RhHDA6-cYFP* were co-transfected into *N. benthamiana* leaves. The empty vectors including nYFP or cYFP were used as negative controls. YFP and mCherry fluorescence was observed by confocal microscopy (Olympus, FV3000, Japan) at 488 nm and 561 nm excitation, respectively. The primers used in BiFC are listed in Supplemental Table S6.

### Co-immunoprecipitation assays

For co-immunoprecipitation assays, 35S::*RhHDA6-GFP* was co-infiltrated with 35S::*ARF18-MYC* into *N. benthamiana* leaves, and co-infiltration of 35S::*GFP* and 35S::*ARF18-MYC* was used as a negative control. Total proteins were extracted from *N. benthamiana* leaves 3 d after infiltration using extraction buffer [50 mM Tris–HCl, pH 7.5, 5 mM EGTA, 10 mM Na_3_VO_4_, 10 mM NaF, 50 mM β-mercaptoethanol, 10 mM DTT, 1 mM PMSF, 5% (v/v) glycerinum and 1% (v/v) protease inhibitor cocktail (Sigma, St Louis, USA)]. The supernatant with different protein combinations was incubated with anti-MYC antibody (Sigma, St Louis, USA) and then analyzed by western blotting using anti-MYC (Abclonal, Wuhan, China) and anti-GFP (Abmart, Shanghai, China) antibodies. The primers used for co-immunoprecipitation assays are listed in Supplemental Table S6.

### Chromatin immunoprecipitation assays

For chromatin immunoprecipitation (ChIP) assays, 1.2 g rose floral buds were cross-linked in polyformaldehyde. The chromatin was sheared to an average length of 500 bp by sonication, and immunoprecipitated with anti-acetylated histone H3K9/K14 (catalogue no. 06-599, Millipore, Billerica, USA; Luo et al., 2012). The probes used for ChIP are listed in Supplemental Table S4. The immunoprecipitated DNA fragments were analyzed by RT-qPCR and the primers used in ChIP assays are listed in Supplemental Table S6.

### Statistical analyses

Statistical analysis was performed using GraphPad Prism (Version 7.0, GraphPad Software Inc., USA: http://www.graphpad.com/). All experimental data were tested with a two-sided Student's *t test* or one-way ANOVA with Tukey's multiple comparisons test.

### Accession numbers

Gene sequence data were deposited to National Center for Biotechnology Information (https://www.ncbi.nlm.nih.gov/geo/) with the following accession numbers: *RhPILS1* (MT799174), *RhARF18* (MT799175) and *RhHDA6* (MT799176).

## Supplemental data

The following supplemental materials are available in the online version of this article.

**Supplemental Figure S1.** Characterization of predicted auxin efflux carrier genes in rose.

**Supplemental Figure S2.** IAA distribution in floral buds of control rose plants and *RhPILS1*-silenced plants.

**Supplemental Figure S3.** Transactivation of the *RhAG* promoter by eight auxin response factors (RhARFs) in *N. benthamiana* leaves.

**Supplemental Figure S4.** Phylogenetic analysis, sequence alignment and subcellular localization of RhARF18.

**Supplemental Figure S5.** Characterization of *RhHDA6*.

**Supplemental Figure S6.** Expression of members of the *RPD3*/*HDA1* family in *RhHDA6*-silenced floral buds.

**Supplemental Figure S7.** Proposed model of auxin-RhARF18/RhHDA6-*RhAG* in petal-stamen homeotic transition.

**Supplemental Table S1.** Auxin efflux carrier gene families from rose transcriptomic database.

**Supplemental Table S2.** Auxin response factors from rose transcriptomic database.

**Supplemental Table S3.** Proteins putatively interacting with RhARF18.

**Supplemental Table S4.** Probes used for ChIP.

**Supplemental Table S5.** *RPD3*/*HDA1* gene family members from rose transcriptomic database.

**Supplemental Table S6.** List of primers used.

## Supplementary Material

kiab130_Supplementary_Data

## Notes

Y.C., D.P., C.-Z.J., S.-S.G., J.G., and N.M. designed research; J.C., Ya.L., Yo.L., Yu.L., C.J., and Y.W. performed research; J.C., Ya.L., Yo.L., Y.C., D.P., P.C., T.X., and N.M. analyzed data; Ya.L., J.G., and N.M. wrote the paper.

The author responsible for distribution of materials integral to the findings presented in this article in accordance with the policy described in the Instructions for Author (https://academic.oup.com/plphys/pages/general-instructions) is: Nan Ma (ma_nan@cau.edu.cn).

## References

[kiab130-B1] Adamowski M, Friml J (2015) PIN-dependent auxin transport: action, regulation, and evolution. Plant Cell 27**:** 20–32 25604445 10.1105/tpc.114.134874 PMC4330589

[kiab130-B2] Barbez E, Kubeš M,, Rolčík J, Béziat C, Pěnčík A, Wang B, Rosquete MR, Zhu J, Dobrev PI, Lee Y, et al (2012) A novel putative auxin carrier family regulates intracellular auxin homeostasis in plants. Nature 485**:** 119–122 22504182 10.1038/nature11001

[kiab130-B3] Bender RL, Fekete ML, Klinkenberg PM, Hampton M, Bauer B, Malecha M,, Lindgren K, Maki JA, Perera MADN, Nikolau BJ, et al (2013) PIN6 is required for nectary auxin response and short stamen development. Plant J 74**:** 893–904 23551385 10.1111/tpj.12184

[kiab130-B4] Benková E, Michniewicz M, Sauer M, Teichmann T, Seifertová D, Jürgens G, Friml J (2003) Local, efflux-dependent auxin gradients as a common module for plant organ formation. Cell 115**:** 591–602 14651850 10.1016/s0092-8674(03)00924-3

[kiab130-B5] Brumos J, Robles LM, Yun J, Vu TC, Jackson S, Alonso JM, Stepanova AN (2018) Local auxin biosynthesis is a key regulator of plant development. Dev Cell 47**:** 306–318.e5 30415657 10.1016/j.devcel.2018.09.022

[kiab130-B6] Chapman EJ, Estelle M (2009) Mechanism of auxin-regulated gene expression in plants. Annu Rev Genet 43**:** 265–285 19686081 10.1146/annurev-genet-102108-134148

[kiab130-B7] Cheng Y, Zhao Y (2007) A role for auxin in flower development. J Integr Plant Biol 49**:** 99–104

[kiab130-B8] Chung Y, Zhu Y, Wu M-F, Simonini S, Kuhn A, Armenta-Medina A, Jin R, Østergaard L, Gillmor CS, Wagner D (2019) Auxin Response Factors promote organogenesis by chromatin-mediated repression of the pluripotency gene *SHOOTMERISTEMLESS*. Nat Commun 10**:** 886 30792395 10.1038/s41467-019-08861-3 PMC6385194

[kiab130-B9] Coen ES, Meyerowitz EM (1991) The war of the whorls: genetic interactions controlling flower development. Nature 353**:** 31–37 1715520 10.1038/353031a0

[kiab130-B10] Drews GN, Bowman JL, Meyerowitz EM (1991) Negative regulation of the Arabidopsis homeotic gene *AGAMOUS* by the *APETALA2* product. Cell 65**:** 991–1002 1675158 10.1016/0092-8674(91)90551-9

[kiab130-B11] Dubois A, Raymond O, Maene M, Baudino S, Langlade NB, Boltz V, Vergne P, Bendahmane M (2010) Tinkering with the C-function: a molecular frame for the selection of double flowers in cultivated roses. PLoS One 5**:** e9288 20174587 10.1371/journal.pone.0009288 PMC2823793

[kiab130-B12] Elena F, Stanislav V, Mugurel IF, Jan P, Jürgen KV (2012) Evolution and structural diversification of PILS putative auxin carriers in plants. Front Plant Sci 3**:** 227 23091477 10.3389/fpls.2012.00227 PMC3470039

[kiab130-B13] Endress PK (2010) The evolution of floral biology in basal angiosperms. Phil Trans R Soc B 365**:** 411–421 20047868 10.1098/rstb.2009.0228 PMC2838258

[kiab130-B14] Friml J (2003) Auxin transport-shaping the plant. Curr Opin Plant Biol 6**:** 7–12 12495745 10.1016/s1369526602000031

[kiab130-B15] Gälweiler L, Guan C, Muller A, Wisman E, Mendgen K, Yephremov A, Palme K (1998) Regulation of polar auxin transport by AtPIN1 in Arabidopsis vascular tissue. Science 282**:** 2226–2230 9856939 10.1126/science.282.5397.2226

[kiab130-B16] Gao Y, Liu Y, Liang Y, Lu J, Jiang C, Fei Z, Jiang C, Ma C, Gao J (2019) *Rosa hybrida* RhERF1 and RhERF4 mediate ethylene‐ and auxin‐regulated petal abscission by influencing pectin degradation. Plant J 99**:** 1159–1171 31111587 10.1111/tpj.14412

[kiab130-B17] Hagen G, Kleinschmidt A, Guilfoyle T (1984) Auxin-regulated gene expression in intact soybean hypocotyl and excised hypocotyl sections. Planta 162**:** 147–153 24254049 10.1007/BF00410211

[kiab130-B18] Han Y-C, Kuang J-F, Chen J-Y, Liu X-C, Xiao Y-Y, Fu C-C, Wang J-N, Wu K-Q, Lu W-J (2016) Banana transcription factor MaERF11 recruits histone deacetylase MaHDA1 and represses the expression of MaACO1 and expansins during fruit ripening. Plant Physiol 171**:** 1070–1084 27208241 10.1104/pp.16.00301 PMC4902611

[kiab130-B19] Hardtke CS, Berleth T (1998) The *Arabidopsis* gene *MONOPTEROS* encodes a transcription factor mediating embryo axis formation and vascular development. EMBO J 17**:** 1405–1411 9482737 10.1093/emboj/17.5.1405 PMC1170488

[kiab130-B20] Heisler MG, Ohno C, Das P, Sieber P, Reddy GV, Long JA, Meyerowitz EM (2005) Patterns of auxin transport and gene expression during primordium development revealed by live imaging of the Arabidopsis inflorescence meristem. Curr Biol 15**:** 1899–1911 16271866 10.1016/j.cub.2005.09.052

[kiab130-B21] Hellens RP, Edwards EA, Leyland NR, Bean S, Mullineaux PM (2000) PGreen: a versatile and flexible binary Ti vector for *Agrobacterium*-mediated plant transformation. Plant Mol Biol 42**:** 819–832 10890530 10.1023/a:1006496308160

[kiab130-B22] Kim HJ, Murai N, Fang DD, Triplett BA (2011) Functional analysis of *Gossypium hirsutum cellulose synthase catalytic subunit 4* promoter in transgenic *Arabidopsis* and cotton tissues. Plant Sci 180**:** 323–332 21421377 10.1016/j.plantsci.2010.10.003

[kiab130-B23] Křeček P, Skůpa P, Libus J, Naramoto S, Tejos R, Friml J, Zažímalová E (2009) The PIN-FORMED (PIN) protein family of auxin transporters. Genome Biol 10**:** 249 20053306 10.1186/gb-2009-10-12-249 PMC2812941

[kiab130-B24] Krizek BA, Fletcher JC (2005) Molecular mechanisms of flower development: an armchair guide. Nat Rev Genet 6**:** 688–698 16151374 10.1038/nrg1675

[kiab130-B25] Krizek BA, Prost V, Macias A (2000) *AINTEGUMENTA* promotes petal identity and acts as a negative regulator of *AGAMOUS*. Plant Cell 12**:** 1357 10948255 10.1105/tpc.12.8.1357 PMC149108

[kiab130-B26] Krogan NT, Hogan K, Long JA (2012) APETALA2 negatively regulates multiple floral organ identity genes in Arabidopsis by recruiting the co-repressor TOPLESS and the histone deacetylase HDA19. Development 139**:** 4180–4190 23034631 10.1242/dev.085407 PMC3478687

[kiab130-B27] Kuhn A, Ramans Harborough S, McLaughlin HM, Natarajan B, Verstraeten I, Friml J, Kepinski S, Østergaard L (2020) Direct ETTIN-auxin interaction controls chromatin states in gynoecium development. eLife 9**:** e51787 32267233 10.7554/eLife.51787 PMC7164952

[kiab130-B28] Lai X, Stigliani A, Vachon G, Carles C, Smaczniak C, Zubieta C, Kaufmann K, Parcy F (2019) Building transcription factor binding site models to understand gene regulation in plants. Mol Plant 12**:** 743–763 30447332 10.1016/j.molp.2018.10.010

[kiab130-B29] Lieberman-Lazarovich M, Yahav C, Israeli A, Efroni I (2019) Deep conservation of cis-element variants regulating plant hormonal responses. Plant Cell 31**:** 2559–2572 31467248 10.1105/tpc.19.00129 PMC6881130

[kiab130-B30] Liu H, Gao Y, Song X, Ma Q, Zhang J, Pei D (2018) A novel rejuvenation approach to induce endohormones and improve rhizogenesis in mature Juglans tree. Plant Methods 14**:** 13 29449873 10.1186/s13007-018-0280-0 PMC5806478

[kiab130-B31] Liu X, Yang S, Zhao M, Luo M, Yu C-W, Chen C-Y, Tai R, Wu K (2014) Transcriptional repression by histone deacetylases in plants. Mol Plant 7**:** 764–772 24658416 10.1093/mp/ssu033

[kiab130-B32] Liu Z, Meyerowitz EM (1995) *LEUNIG* regulates *AGAMOUS* expression in Arabidopsis flowers. Development 121**:** 975 7743940 10.1242/dev.121.4.975

[kiab130-B33] Luo M, Yu C-W, Chen F-F, Zhao L, Tian G, Liu X, Cui Y, Yang J-Y, Wu K (2012) Histone deacetylase HDA6 is functionally associated with AS1 in repression of *KNOX* genes in Arabidopsis. PLoS Genet 8**:** e1003114 23271976 10.1371/journal.pgen.1003114 PMC3521718

[kiab130-B34] Ma N, Chen W, Fan T, Tian Y, Zhang S, Zeng D, Li Y (2015) Low temperature-induced DNA hypermethylation attenuates expression of *RhAG*, an *AGAMOUS* homolog, and increases petal number in rose (*Rosa hybrid*a). BMC Plant Biol 15**:** 237 26438149 10.1186/s12870-015-0623-1 PMC4595006

[kiab130-B35] Ma N, Xue J, Li Y, Liu X, Dai F, Jia W, Luo Y, Gao J (2008) *Rh-PIP2;1*, a rose aquaporin gene, is involved in ethylene-regulated petal expansion. Plant Physiol 148**:** 894–907 18715962 10.1104/pp.108.120154 PMC2556823

[kiab130-B36] Okada K, Ueda J, Komaki MK, Bell CJ, Shimura Y (1991) Requirement of the auxin polar transport system in early stages of Arabidopsis floral bud formation. Plant Cell 3**:** 677–684 12324609 10.1105/tpc.3.7.677 PMC160035

[kiab130-B37] Pei H, Ma N, Chen J, Zheng Y, Tian J, Li J, Zhang S, Fei Z, Gao J (2013a) Integrative analysis of miRNA and mRNA profiles in response to ethylene in rose petals during flower opening. PLoS One 8**:** e64290 23696879 10.1371/journal.pone.0064290 PMC3655976

[kiab130-B38] Pei H, Ma N, Tian J, Luo J, Chen J, Li J, Zheng Y, Chen X, Fei Z, Gao J (2013b) An NAC transcription factor controls ethylene-regulated cell expansion in flower petals. Plant Physiol 163**:** 775–791 23933991 10.1104/pp.113.223388 PMC3793057

[kiab130-B39] Petrasek J (2006) PIN proteins perform a rate-limiting function in cellular auxin efflux. Science 312**:** 914–918 16601150 10.1126/science.1123542

[kiab130-B40] Raymond O, Gouzy J, Just J, Badouin H, Verdenaud M, Lemainque A, Vergne P, Moja S, Choisne N, Pont C, et al (2018) The Rosa genome provides new insights into the domestication of modern roses. Nat Genet 50**:** 772–777 29713014 10.1038/s41588-018-0110-3 PMC5984618

[kiab130-B41] Reinhardt D, Mandel T, Kuhlemeier C (2000) Auxin regulates the initiation and radial position of plant lateral organs. Plant Cell 12**:** 507–518 10760240 10.1105/tpc.12.4.507 PMC139849

[kiab130-B42] Sessions A, Nemhauser JL, McColl A, Roe JL, Feldmann KA, Zambryski PC (1997) *ETTIN* patterns the Arabidopsis floral meristem and reproductive organs. Development 124**:** 4481–4491 9409666 10.1242/dev.124.22.4481

[kiab130-B43] Sessions RA, Zambryski PC (1995) *Arabidopsis* gynoecium structure in the wild and in *ettin* mutants. Development 121**:** 1519 7789281 10.1242/dev.121.5.1519

[kiab130-B44] Shen C, Yue R, Sun T, Zhang L, Xu L, Tie S, Wang H, Yang Y (2015) Genome-wide identification and expression analysis of auxin response factor gene family in *Medicago truncatula*. Front Plant Sci 6: 73 10.3389/fpls.2015.00073 PMC4338661 25759704

[kiab130-B45] Shen J, Zhang Y, Ge D, Wang Z, Song W, Gu R, Che G, Cheng Z, Liu R, Zhang X (2019) CsBRC1 inhibits axillary bud outgrowth by directly repressing the auxin efflux carrier *CsPIN3* in cucumber. Proc Natl Acad Sci USA 116**:** 17105–17114 31391306 10.1073/pnas.1907968116 PMC6708385

[kiab130-B46] Theißen G, Melzer R, Rümpler F (2016) MADS-domain transcription factors and the floral quartet model of flower development: linking plant development and evolution. Development 143**:** 3259–3271 27624831 10.1242/dev.134080

[kiab130-B47] Theißen G, Rümpler F (2018) Evolution of floral organ identity. *In* Nuno de la Rosa L, Müller G, eds, Evolutionary Developmental Biology. Springer International Publishing, Cham, pp 1–17

[kiab130-B48] van Berkel K, de Boer RJ, Scheres B, ten Tusscher K (2013) Polar auxin transport: models and mechanisms. Development 140**:** 2253–2268 23674599 10.1242/dev.079111

[kiab130-B49] Weigel D, Meyerowitz EM (1994) The ABCs of floral homeotic genes. Cell 78**:** 203–209 7913881 10.1016/0092-8674(94)90291-7

[kiab130-B50] Wisniewska J (2006) Polar PIN localization directs auxin flow in plants. Science 312**:** 883–883 16601151 10.1126/science.1121356

[kiab130-B51] Wu M-F, Yamaguchi N, Xiao J, Bargmann B, Estelle M, Sang Y, Wagner D (2015) Auxin-regulated chromatin switch directs acquisition of flower primordium founder fate. eLife 4**:** e09269 26460543 10.7554/eLife.09269 PMC4600763

[kiab130-B52] Xu Y, Prunet N, Gan E, Wang Y, Stewart D, Wellmer F, Huang J, Yamaguchi N, Tatsumi Y, Kojima M, et al (2018) SUPERMAN regulates floral whorl boundaries through control of auxin biosynthesis. EMBO J 37**:** e97499 29764982 10.15252/embj.201797499 PMC5983216

[kiab130-B53] Yamaguchi N, Huang J, Tatsumi Y, Abe M, Sugano SS, Kojima M, Takebayashi Y, Kiba T, Yokoyama R, Nishitani K, et al (2018) Chromatin-mediated feed-forward auxin biosynthesis in floral meristem determinacy. Nat Commun 9**:** 5290 30538233 10.1038/s41467-018-07763-0 PMC6289996

[kiab130-B54] Yamaguchi N, Huang J, Xu Y, Tanoi K, Ito T (2017) Fine-tuning of auxin homeostasis governs the transition from floral stem cell maintenance to gynoecium formation. Nat Commun 8**:** 1125 29066759 10.1038/s41467-017-01252-6 PMC5654772

[kiab130-B55] Yamaguchi N, Wu M-F, Winter CM, Berns MC, Nole-Wilson S, Yamaguchi A, Coupland G, Krizek BA, Wagner D (2013) A molecular framework for auxin-mediated initiation of flower primordia. Dev Cell 24**:** 271–282 23375585 10.1016/j.devcel.2012.12.017

[kiab130-B56] Yan W, Chen D, Kaufmann K (2016) Molecular mechanisms of floral organ specification by MADS domain proteins. Curr Opin Plant Biol 29**:** 154–162 26802807 10.1016/j.pbi.2015.12.004

[kiab130-B57] Yanofsky MF, Ma H, Bowman JL, Drews GN, Feldmann KA, Meyerowitz EM (1990) The protein encoded by the Arabidopsis homeotic gene *agamous* resembles transcription factors. Nature 346**:** 35–39 1973265 10.1038/346035a0

[kiab130-B58] Yu C-W, Liu X, Luo M, Chen C, Lin X, Tian G, Lu Q, Cui Y, Wu K (2011) HISTONE DEACETYLASE6 interacts with FLOWERING LOCUS D and regulates flowering in Arabidopsis. Plant Physiol 156**:** 173–184 21398257 10.1104/pp.111.174417 PMC3091038

[kiab130-B59] Zhang S, Feng M, Chen W, Zhou X, Lu J, Wang Y, Li Y, Jiang C-Z, Gan S-S, Ma N, et al (2019) In rose, transcription factor PTM balances growth and drought survival via PIP2;1 aquaporin. Nat Plants 5**:** 290–299 30833710 10.1038/s41477-019-0376-1

[kiab130-B60] Zhao Y (2010) Auxin biosynthesis and its role in plant development. Annu Rev Plant Biol 61**:** 49–64 20192736 10.1146/annurev-arplant-042809-112308 PMC3070418

