# Supplementary material for: AUXIN RESPONSE FACTOR 18–HISTONE DEACETYLASE 6 module regulates floral organ identity in rose (Rosa hybrida)
Source: Plant Physiol. 2021 Mar 17;186(2):1074–87. doi: 10.1093/plphys/kiab130 (PMC8195501; doi:10.1093/plphys/kiab130)
Supplement: kiab130_Supplementary_Data [file kiab130_supplementary_data.zip › pp.00103.2021-s08.pdf]

**Short title: ARF18-HDA6 module controls floral organ identity**

# AUXIN RESPONSE FACTOR 18–HISTONE DEACETYLASE 6 module regulates floral organ identity in rose (*Rosa hybrida*)

Jiwei Chen[1,a], Yang Li[1,a], Yonghong Li[2], Yuqi Li[1], Yi Wang[1], Chuyan Jiang[1], Patrick Choisy[3], Tao Xu[3], Youming Cai[4], Dong Pei[5], Cai-Zhong Jiang[6,7], Su-Sheng Gan[8], Junping Gao[1], Nan Ma[1,b]

[1]State Key Laboratory of Agrobiotechnology, Beijing Key Laboratory of Development and Quality Control of Ornamental Crops, Department of Ornamental Horticulture, College of Horticulture, China Agricultural University, Beijing 100193, China

[2]School of Applied Chemistry and Biotechnology, Shenzhen Polytechnic, Shenzhen, Guangdong 518055, China

[3]LVMH Recherche, 185 avenue de Verdun F-45800 St Jean de Braye, France

[4]Shanghai Academy of Agricultural Sciences, Shanghai 201403, China

[5]State Key Laboratory of Tree Genetics and Breeding, Research Institute of Forestry, Chinese Academy of Forestry, Beijing 100091, China

[6]Crop Pathology and Genetic Research Unit, United States Department of Agriculture, Agricultural Research Service, Davis, CA, USA

[7]Department of Plant Sciences, University of California, Davis, CA, USA

[8]Plant Biology Section, School of Integrative Plant Science, College of Agriculture and Life Sciences, Cornell University, Ithaca, NY, USA

[a] J.C. and Y.L. contributed equally to this work.

[b] To whom the correspondence should be addressed.

Professor Nan Ma, Email: ma_nan@cau.edu.cn

# SUPPLEMENTAL FIGURES AND LEGENDS

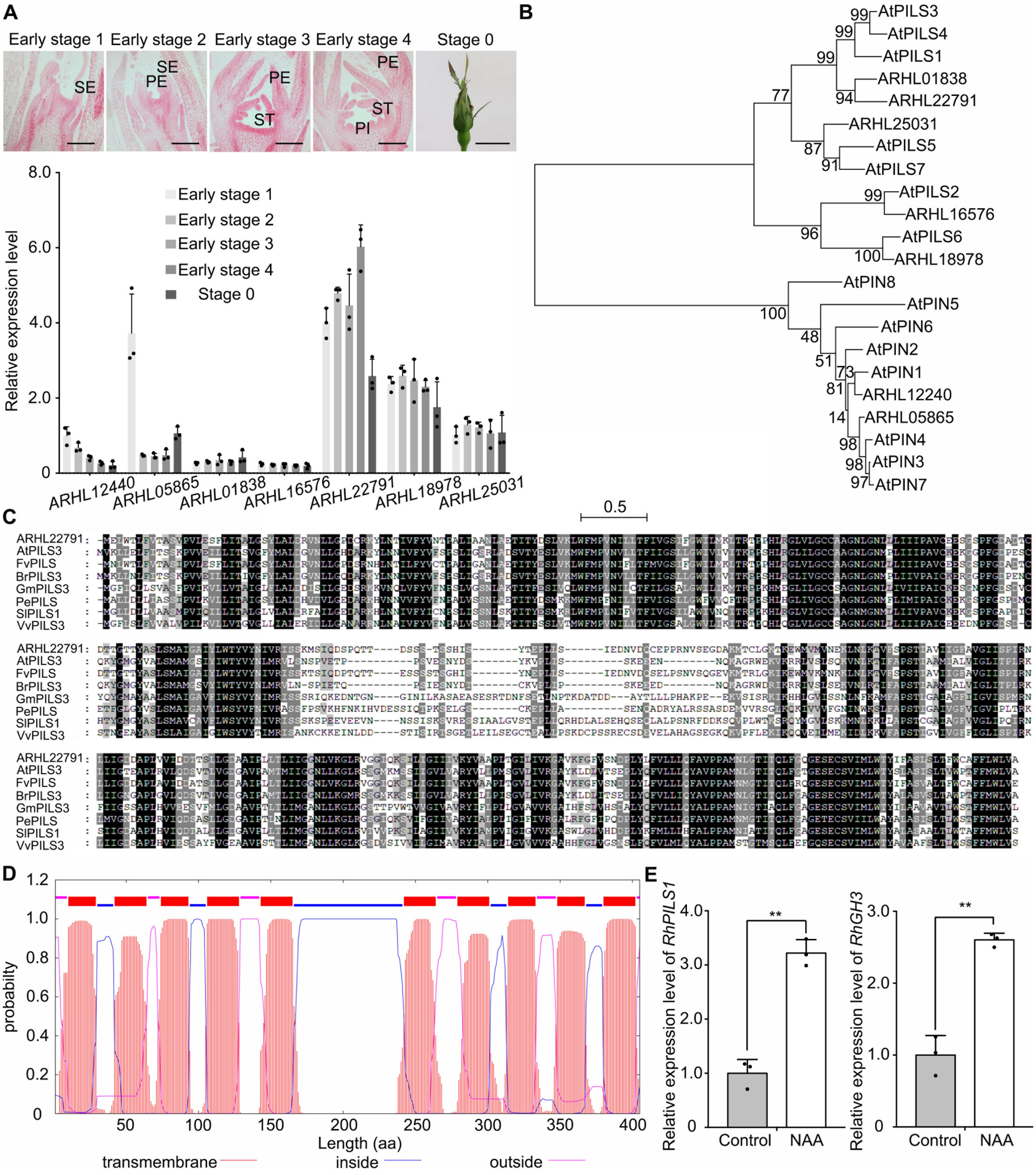


**Supplemental Figure S1. Characterization of predicted auxin efflux carrier genes in rose.**

**(A)** Quantitative RT-PCR of seven predicted auxin efflux carrier genes during floral organogenesis in rose. Floral organogenesis stages were defined as follows: early stage 1, sepal primordia initiation; early stage 2, petal primordia initiation; early stage 3, stamen primordia initiation; early stage 4, pistil primordia initiation; Stage 0, bud revealed. *RhUBI2* was used as an internal control. The mean values ± SD are shown

from three biological replicates (n = 3). SE, sepal; PE, petal; ST, stamen; PI, pistil. Scale bars, 200 μm for early stage 1 - 4 and 1 cm for stage 0.

**(B)** Phylogenetic analysis of auxin efflux genes of rose and *A. thaliana*. Only the conserved domains were used and all positions with less than 80% site coverage were eliminated. The phylogenetic tree was constructed using the Maximum Likelihood method based on LG + G model within MEGA software (version X). The percentage of trees in which the associated taxa clustered together is shown next to the branches. The tree is drawn to scale, with branch lengths measured in the number of substitutions per site.

**(C)** Alignment of putative amino acid sequences of PILS protein from rose and seven other species: AtPILS3 (AAM62517), *A. thaliana*; FvPILS (XP_004300096), *Fragaria vesca* subsp. *Vesca*; BrPILS3 (XP_009106321), *Brassica rapa*; GmPILS3 (XP_006599267), *Glycine max*; PePILS (XP_011034783), *Populus euphratica*; SlPILS1 (XP_004253114), *Solanum lycopersicum*; VvPILS3 (XP_010649328), *Vitis vinifera*. The conserved degree of amino acid sites increased with color deepening under Four-level shadow mode within GeneDoc software. Amino acids that are similar in 100% of aligned sequences are shaded black, 60%–80% grey.

**(D)** The transmembrane domains of RhPILS1 were predicted online by TMHMM server 2.0 (https://services.healthtech.dtu.dk/service.php?TMHMM-2.0).

**(E)** Quantitative RT-PCR of *RhPILS1* and *RhGH3* in response to auxin. *RhUBI2* was used as an internal control. The mean values ± SD are shown from three biological replicates (n = 3). Asterisks indicate statistically significant differences (two-sided Student's *t*-test; **, $P < 0.01$).

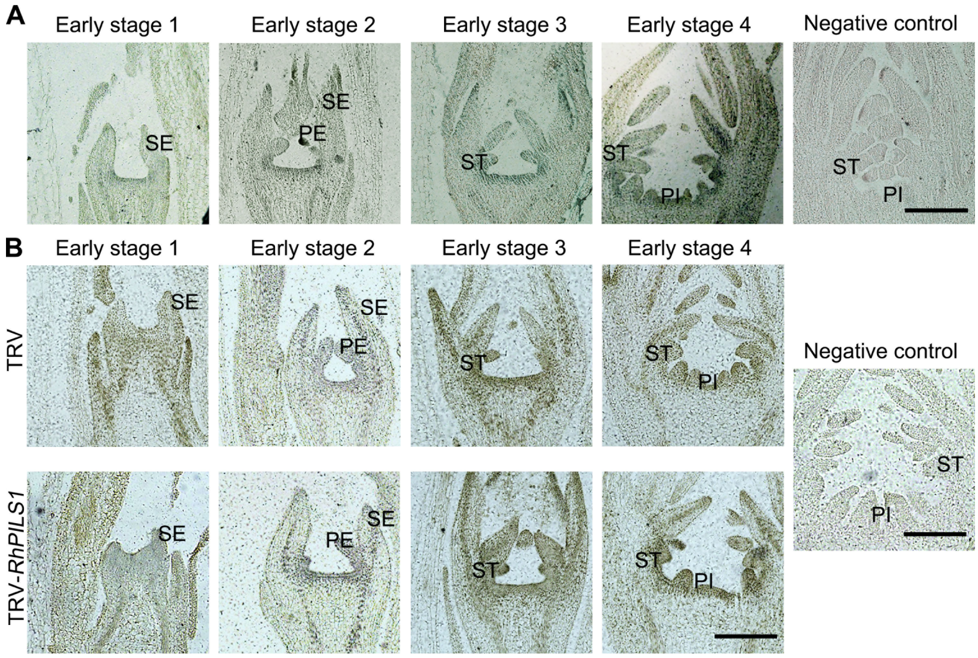


**Supplemental Figure S2. IAA distribution in floral buds of control rose plants and *RhPILS1*-silenced plants.**

**(A)** IAA level in buds during floral organ development. Free IAA was monitored by immuno-gold localization in floral buds. The floral buds at early stage 4 without primary antibody were used as a negative control. SE, sepal; PE, petal; ST, stamen; PI, pistil. Scale bars, 200 µm.

**(B)** Silencing of *RhPILS1* altered IAA distribution in floral buds. IAA distribution was monitored by immuno-gold localization in TRV control and *RhPILS1*-silenced floral buds. The floral buds of TRV-control at early stage 4 without primary antibody were used as a negative control. At least 10 TRV and 10 TRV-*RhPILS1* plants were used. Scale bars, 200 µm.

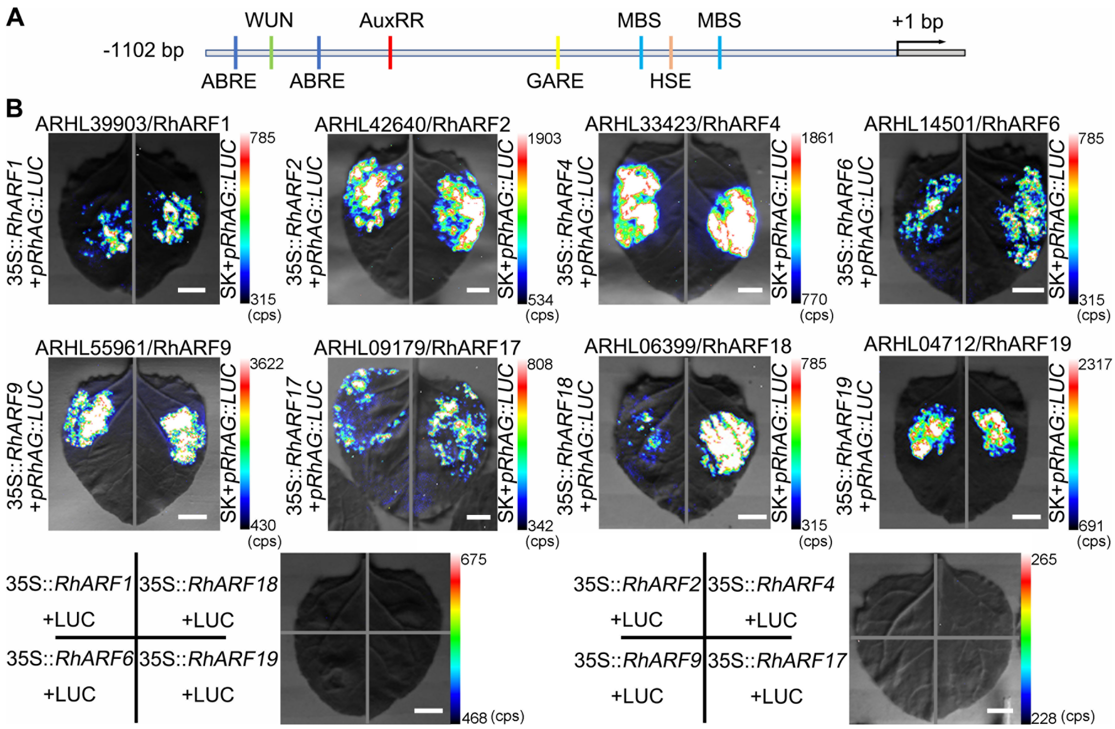


**Supplemental Figure S3. Transactivation of the *RhAG* promoter by eight auxin responsive factors (RhARFs) in *N. benthamiana* leaves.**

**(A)** Schematic representation of *cis*-elements in the *RhAG* promoter. The *cis*-elements were predicted online by PlantCARE (http://bioinformatics.psb.ugent.be/webtools/plantcare/html/).

**(B)** Transactivation of the *RhAG* promoter by *RhARFs* in *N. benthamiana* leaves.

The p*RhAG*::*LUC* (*luciferase*) construct was co-infiltrated with empty vector (as a control) or co-infiltrated with each single 35S::*RhARFs* in *N. benthamiana* leaves. Bottom panel, LUC empty vector was co-infiltrated with each single 35S::*RhARFs* as another negative controls. The experiments were performed independently three times, and similar results were obtained. For each *RhARF*, a representative image of an *N. benthamiana* leaf 3 d after infiltration is shown. Scale bars, 1 cm.

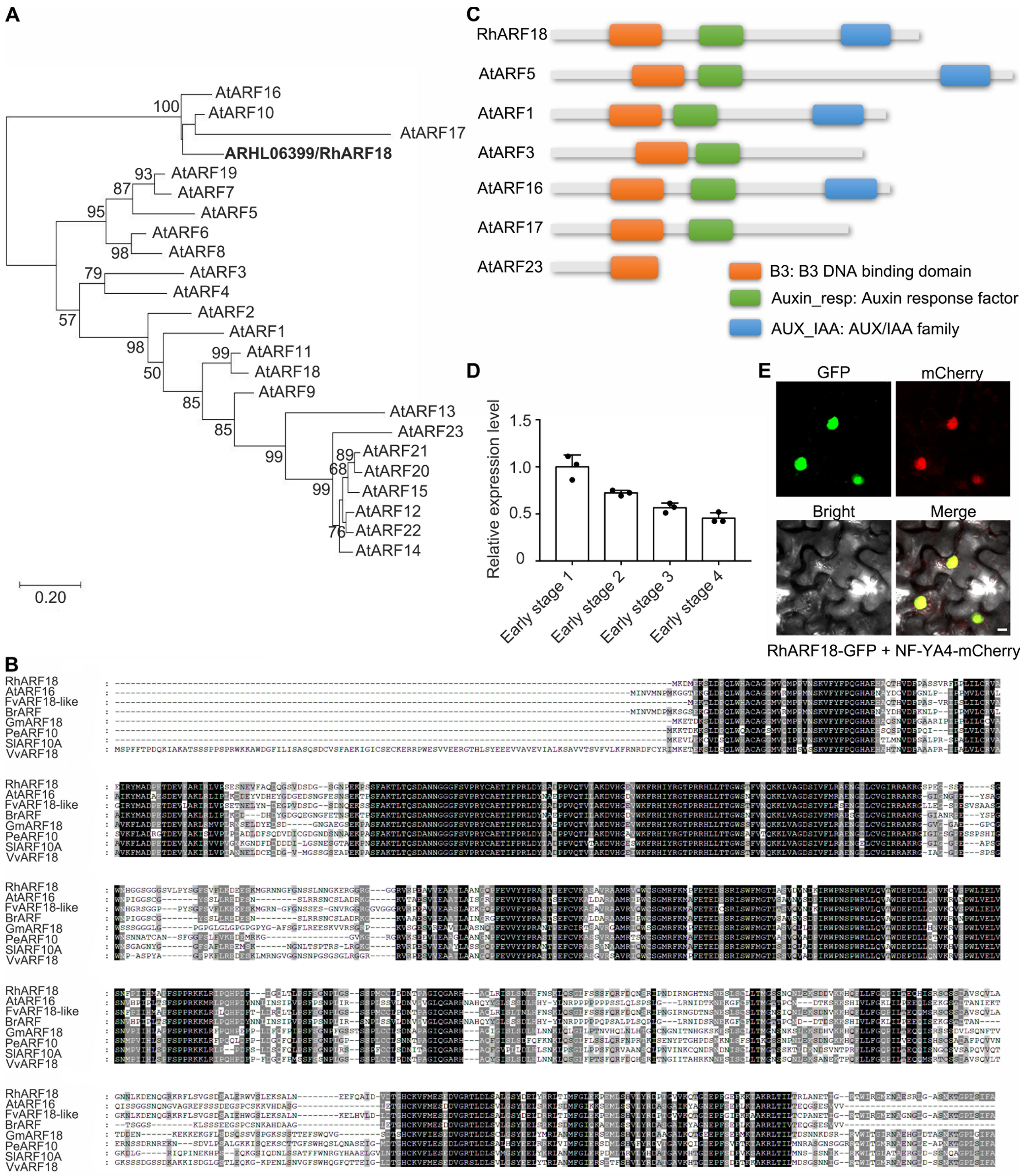


**Supplemental Figure S4. Phylogenetic analysis, sequence alignment and subcellular localization of RhARF18.**

**(A)** Phylogenetic analysis of RhARF18 and ARFs from *A. thaliana*. Only the conserved domains were used and all positions with less than 80% site coverage were eliminated. The phylogenetic tree was constructed using the Maximum Likelihood method based on WAG + G model within MEGA software (version X). The percentage of trees in which the associated taxa clustered together is shown next to the branches. The tree is drawn to scale, with branch lengths measured in the number of substitutions per site.

**(B)** Alignment of putative amino acid sequences of RhARF18 protein and eight other species: AtARF16 (NP_567841.1), *A. thaliana*; FvARF18-like (XP_011459440.1), *F.*

*vesca* subsp. *Vesca*; BrARF (RIA04857.1), *B. rapa*; GmARF18 (XP_003540185.1), *G. max*; PeARF10 (XP_011040890.1), *P. euphratica*; SlARF10A (NP_001234796.2), *S. lycopersicum*; VvARF18 (RVW38797.1), *V. vinifera*. The conserved degree of amino acid sites increased with color deepening under Four-level shadow mode within GeneDoc software. Amino acids that are similar in 100% of aligned sequences are shaded black, 60%–80% grey.

**(C)** Conserved domains of RhARF18 and 6 ARFs from *A. thaliana*. The protein domains were predicted online by Pfam 33.1 (http://pfam.xfam.org) and NCBI conserved domain search (https://www.ncbi.nlm.nih.gov/Structure/cdd/wrpsb.cgi).

**(D)** Quantitative RT-PCR of *RhARF18* during floral organogenesis in rose. Floral organogenesis stages were defined as follows: early stage 1, sepal primordia initiation; early stage 2, petal primordia initiation; early stage 3, stamen primordia initiation; early stage 4, pistil primordia initiation. *RhUBI2* was used as an internal control. The mean values ± SD are shown from three biological replicates (n = 3).

**(E)** Subcellular localization of RhARF18-GFP heterologously expressed in *N. benthamiana* leaves. For *N. benthamiana* leaves, *RhARF18-GFP* was co-infiltrated with the nuclear marker *NF-YA4-mCherry*. Green and red fluorescence were visualized by confocal microscopy 3 d after infiltration. The experiment was performed independently three times, and representative results are shown. Scale bar, 10 μm.

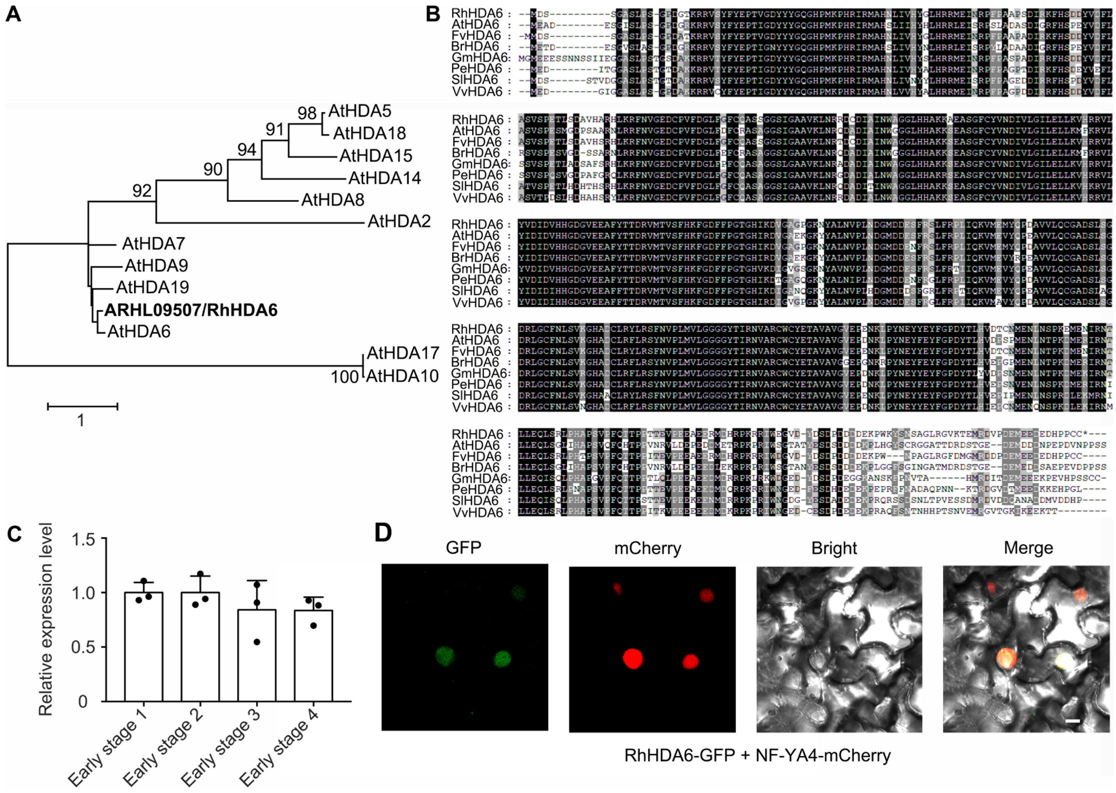


**Supplemental Figure S5. Characterization of *RhHDA6*.**

**(A)** Phylogenetic analysis of ARHL09507 (RhHDA6) with *RPD3/HDA1* gene family of histone deacetylase proteins from *A. thaliana*. Only the conserved domains were used and all positions with less than 80% site coverage were eliminated. The phylogenetic tree was constructed using the Maximum Likelihood method based on LG + G model within MEGA software (version X). The percentage of trees in which the associated taxa clustered together is shown next to the branches. The tree is drawn to scale, with branch lengths measured in the number of substitutions per site.

**(B)** Alignment of putative amino acid sequences of HDA6 proteins from rose and seven other species: AtHDA6 (NP_201116.1), *A. thaliana*; FvHDA6 (XP_004299132.1), *F. vesca* subsp. *Vesca*; BrHDA6 (XP_009150370.1), *B. rapa*; GmHDA6 (XP_003525556.1), *G. max*; PeHDA6 (XP_011046214.1), *P. euphratica*; SlHDA6 (NP_001352081.1), *S. lycopersicum*; VvHDA6 (XP_010663108.1), *V. vinifera*. The conserved degree of amino acid sites increased with color deepening under Four-level shadow mode within GeneDoc software. Amino acids that are similar in 100% of aligned sequences are shaded black, 60%–80% grey.

**(C)** Quantitative RT-PCR of *RhHDA6* during floral organogenesis in rose. Floral organogenesis stages were defined as follows: early stage 1, sepal primordia initiation; early stage 2, petal primordia initiation; early stage 3, stamen primordia initiation; early

stage 4, pistil primordia initiation. *RhUBI2* was used as an internal control. The mean values ± SD are shown from three biological replicates (n = 3).

**(D)** Subcellular localization of RhHDA6-GFP heterologously expressed in *N. benthamiana* leaves. For *N. benthamiana* leaves, *RhHDA6-GFP* was co-infiltrated with the nuclear marker *NF-YA4-mCherry*. Green and red fluorescence were visualized by confocal microscopy 3 d after infiltration. The experiment was performed independently three times, and representative results are shown. Scale bar, 10 µm.

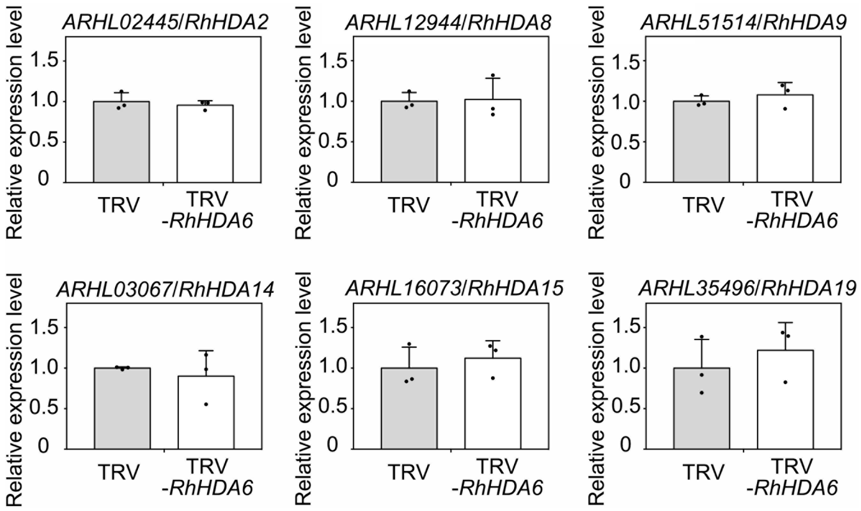


**Supplemental Figure S6. Expression of members of the *RPD3*/*HDA1* family in *RhHDA6*-silenced floral buds.**

Quantitative RT-PCR of *RPD3*/*HDA1* gene family members in TRV and TRV-*RhHDA6* floral buds. At least 8 floral buds were mixed as one biological sample of early stage 4. The mean values ± SD from three biological replicates (n = 3) are shown. *RhUBI2* was used as an internal control.

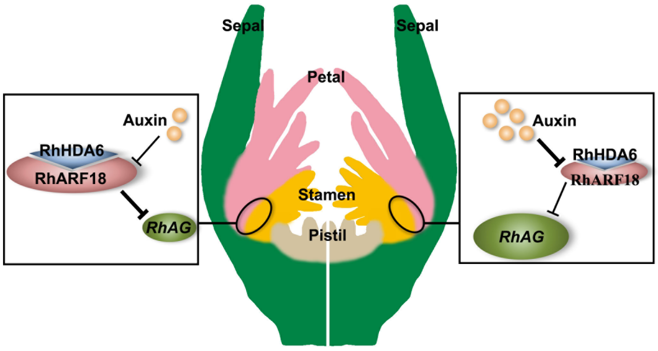


**Supplemental Figure S7. Proposed model of auxin-RhARF18/RhHDA6-*RhAG* in petal-stamen homeotic transition.**

Left, normal auxin level keeps RhARF18 at a proper level and thus restricts *RhAG* level, leading to normal development of petals and stamens. Right, silencing of RhPILS1 results in elevated auxin level, which attenuates RhARF18 expression. Consequently, expression of *RhAG* is released and triggers homeotic transformation from petals to stamens.
