# Supplementary material for: AUXIN RESPONSE FACTOR 18–HISTONE DEACETYLASE 6 module regulates floral organ identity in rose (Rosa hybrida)
Source: Plant Physiol. 2021 Mar 17;186(2):1074–87. doi: 10.1093/plphys/kiab130 (PMC8195501; doi:10.1093/plphys/kiab130)
Supplement: kiab130_Supplementary_Data [file kiab130_supplementary_data.zip › pp.00103.2021-s05.docx]

Table S4. Probes used for ChIP.

| **Probe ID** | **upstream to the ATG of *RhAG*** | **Length (bp)** |
| --- | --- | --- |
| Aux-RR core | -853 to -847 | 7 |
| P1 | -861 to -644 | 218 |
| P2 | -662 to -465 | 198 |
| P3 | -484 to -286 | 199 |
| P4 | -318 to -172 | 147 |
| P5 | -195 to -1 | 195 |

***RhAG* promoter (1102 bp)**

The Aux-RR core was highlighted by red and the first codon was highlighted by yellow.

TGTCTTTACGACCCTCCAATCAGAAGAGAGGAAATTTTCAATCTTTTCCAAAATTGCCCCTGCTCCCTGAAGTGCAATACCCAAAACACTCCCCTGCTGGGCATTGATTGGCCCTCCCCACCACGTGTCCGTGCGGGTGCCCTACGCAAGATTCTCTCCTCTTCCTCCTCTATATCCCATCAATCATTCACAGGTAAAAGAATTAATGTATATCTTTGATAAAATAAATGAGTAAAAACTGGACGGTTGATGGACCCTGAATCAAATGAGTGATTCCCATGGTTTCTCTTTACTTTTGCTTCAGGTCCAATACAAAATGTATTATACCACTTACATTTCCTTGTAATAATAACTAAAGTCATATACATTAAAAATTGAAGCCAAGTTTTGGAAATTAGTATAATGGAGGAAAGATCGTTTTTCCCTTTATAAATACCCTTCTCTGAGTCCCCCTTGCTTCCATTTTCTGCATATCTTCTTGTTTAGATTGTGGAAAAGAAAAGAAGTCTAAGAAAAAACCCAAAAGGTAGAAACCTCTCTGTTTCTTTCATCATCTCCATCTTCTCTTTGTTTTCTTATCTGGGTATTGATCAAAATACTCAAAACACCATCTGGTAATCCAATCTTCAGTGCCTCACTTTTTTAGTCCAATTCATGTTTTTGACTTTTGAGTACTCCACAGCTAGCTAGATCAGCAACTGCTTGATGTTTGTTGTCAAAGAACCCAAAAAGCAATGAACTTAAAATTTTTTAGTCCATCAGTTCACAATTTCTTTTAGTCCAATTCATGTTTTTAGCTCCACAGCTAGATCAGCAACTGTACTTTGATGTTTGTTGTCAAAGAACCCAAAGTGCAATGAACTTATTAACTCTCAGTCCATCAGTTCACAATTTCTTGAGCTAGTTAGCTAGTTGTAGTGTAACACCATGCTTTTCTTGATCAAAGCTAGATAGGGGAGATTAGTACTATTTAGAAACCGTCTTTTGATTTTCTAATTTGTACATAAAGTTTGATCCTTTTTTGCTTGATTGATGATTCTCATTGTACATTGGCCTTTTTTTTTTCTTTCCCCTACTTGATTGATGACTCTCAGCTGCAACTATG

P1: GACGGTTGATGGACCCTGAATCAAATGAGTGATTCCCATGGTTTCTCTTTACTTTTGCTTCAGGTCCAATACAAAATGTATTATACCACTTACATTTCCTTGTAATAATAACTAAAGTCATATACATTAAAAATTGAAGCCAAGTTTTGGAAATTAGTATAATGGAGGAAAGATCGTTTTTCCCTTTATAAATACCCTTCTCTGAGTCCCCCTTGCTT

P2:

CTCTGAGTCCCCCTTGCTTCCATTTTCTGCATATCTTCTTGTTTAGATTGTGGAAAAGAAAAGAAGTCTAAGAAAAAACCCAAAAGGTAGAAACCTCTCTGTTTCTTTCATCATCTCCATCTTCTCTTTGTTTTCTTATCTGGGTATTGATCAAAATACTCAAAACACCATCTGGTAATCCAATCTTCAGTGCCTCAC

P3:

TCCAATCTTCAGTGCCTCACTTTTTTAGTCCAATTCATGTTTTTGACTTTTGAGTACTCCACAGCTAGCTAGATCAGCAACTGCTTGATGTTTGTTGTCAAAGAACCCAAAAAGCAATGAACTTAAAATTTTTTAGTCCATCAGTTCACAATTTCTTTTAGTCCAATTCATGTTTTTAGCTCCACAGCTAGATCAGCAA

P4:

TTCATGTTTTTAGCTCCACAGCTAGATCAGCAACTGTACTTTGATGTTTGTTGTCAAAGAACCCAAAGTGCAATGAACTTATTAACTCTCAGTCCATCAGTTCACAATTTCTTGAGCTAGTTAGCTAGTTGTAGTGTAACACCATGC

P5:

GCTAGTTGTAGTGTAACACCATGCTTTTCTTGATCAAAGCTAGATAGGGGAGATTAGTACTATTTAGAAACCGTCTTTTGATTTTCTAATTTGTACATAAAGTTTGATCCTTTTTTGCTTGATTGATGATTCTCATTGTACATTGGCCTTTTTTTTTTCTTTCCCCTACTTGATTGATGACTCTCAGCTGCAACT
